# High-order interactions distort the functional landscape of microbial consortia

**DOI:** 10.1371/journal.pbio.3000550

**Published:** 2019-12-12

**Authors:** Alicia Sanchez-Gorostiaga, Djordje Bajić, Melisa L. Osborne, Juan F. Poyatos, Alvaro Sanchez

**Affiliations:** 1 Department of Ecology & Evolutionary Biology, Yale University, New Haven, Connecticut, United States of America; 2 Microbial Sciences Institute, Yale University, West Haven, Connecticut, United States of America; 3 The Rowland Institute at Harvard, Harvard University, Cambridge, Massachusetts, United States of America; 4 Biological Design Center, Boston University, Boston, Massachusetts, United States of America; 5 Logic of Genomic Systems Laboratory, Spanish National Biotechnology Centre (CNB-CSIC), Madrid, Spain; Fred Hutchinson Cancer Research Center, UNITED STATES

## Abstract

Understanding the link between community composition and function is a major challenge in microbial population biology, with implications for the management of natural microbiomes and the design of synthetic consortia. Specifically, it is poorly understood whether community functions can be quantitatively predicted from traits of species in monoculture. Inspired by the study of complex genetic interactions, we have examined how the amylolytic rate of combinatorial assemblages of six starch-degrading soil bacteria depend on the separate functional contributions from each species and their interactions. Filtering our results through the theory of biochemical kinetics, we show that this simple function is additive in the absence of interactions among community members. For about half of the combinatorially assembled consortia, the amylolytic function is dominated by pairwise and higher-order interactions. For the other half, the function is additive despite the presence of strong competitive interactions. We explain the mechanistic basis of these findings and propose a quantitative framework that allows us to separate the effect of behavioral and population dynamics interactions. Our results suggest that the functional robustness of a consortium to pairwise and higher-order interactions critically affects our ability to predict and bottom-up engineer ecosystem function in complex communities.

## Abstract 2

Can we predict the function of a microbial consortium? This study shows that even a simple function can exhibit substantial complexity and be dominated by complex interactions, illustrating the important challenges that arise when trying to design synthetic microbial consortia from first principles.

## Introduction

Microbial communities carry out critical biochemical functions throughout the biosphere: from nitrogen fixation and photosynthesis to the recycling of nutrients and the decomposition of organic matter [1,2]. In host-associated communities, the metabolic activity of the microbiota can also profoundly affect the host’s health, modulating life-history traits such as flowering timing in plants [3,4] or the life span and reproductive behavior of animals [5,6]. In more applied settings, microbial consortia are being designed for the processing of undesirable materials into valuable products [7–11], to protect valuable crops from pathogens [12,13] and other stressors [14,15], or to increase crop yields [16]. The specific effects that microbial communities have on their environment or their hosts can be termed the “functions” of these communities. These functions depend on community composition—i.e., on which species are present and their abundance. Thus, manipulating community composition to accomplish desirable functional outcomes has become a major goal in fields as diverse as medicine, environmental engineering, and biotechnology [15].

To accomplish this goal, it is imperative to develop a predictive understanding of the relationship between microbial community composition and function [17–21]. This is widely recognized as one of the main challenges in the field [7,17,18,22,23], but many fundamental questions remain. Importantly, it is still unclear to what extent one can predict the function of a large multispecies community from low-dimensional information, such as the functional contributions of single species and their pairwise interactions [24–28]. The answer to this question has important implications: if predicting community function in such a bottom-up manner were generally feasible, this would encourage synthetic approaches to designing complex communities in a rational manner, by mixing and matching components with known functional traits [7,18,27,29]. However, the contribution of a given species or pair of species to a community function may also depend on the presence or absence of other taxa, for instance, through ecological interactions that modulate species abundance, or by modulating the expression of functionally relevant genes. This can easily lead to higher-than-pairwise functional interactions. If community functions were generally complex and enriched in nonlinear or high-order functional interactions, bottom-up prediction would be significantly more challenging, and top-down approaches such as community-level selection [30] might be a more viable strategy for the manipulation and design of complex consortia [4,14,30–34].

The crux of the problem is thus the contribution of pairwise and high-order interactions (HOIs) to community functions. In spite of a growing appreciation of the role that HOIs may play in community assembly [28,35–40], we still know little about their quantitative contribution to specific processes and community-level functions [6,20]. To tackle this question, one would need to disentangle the functional contributions of all of the single species in isolation from the effects of pairwise and higher-order interactions. This is a notoriously challenging problem, particularly for complex functions for which “first principles” mechanistic models are hard to produce [41].

A similar problem has been encountered many times before in other areas of biology, most notably in the study of genetic interactions among mutations in fitness landscapes [42–44]. To detect interactions, one compares the quantitative phenotype of multiple mutations with the expectation from a null model that assumes that the effects of mutations are independent. In practice, this often is taken to mean that the phenotypic effects of mutations add up [45–47], and the difference between the measured phenotype and the null model prediction is thus attributed to genetic interactions [41–47]. When the interaction between a pair of mutations is affected by the presence of a third mutation, a third-order interaction is found. In recent years, strategies that are inspired by the study of complex interactions on fitness landscapes have been deployed to detect interactions in other complex biological systems, e.g., between transcription factors in combinatorial gene regulation [48,49] or among multiple drugs in antibiotic or cancer drug cocktails [50,51].

Applied to microbial communities, this strategy would involve reconstituting all possible combinatorial subcommunities (i.e., every possible monoculture as well as every possible pairwise coculture, three-species coculture, four-member coculture, etc.), measuring their function, and then comparing this measurement to the prediction of a null model [6,20]. Defining a null model that captures the absence of interactions is therefore critical to unambiguously establish the contribution of both pairwise interactions and HOIs to community-level functions. Yet, for complex functions that are also often inherently nonlinear and depend in poorly understood ways on the molecular interplay between hosts and microbes, it is often not obvious what that null model should be.

To circumvent this problem, we set out to study a simple community-level function that can be quantitatively modeled from the bottom up from mechanistic biochemical principles. The quantitative characterization of this function, combined with theory borrowed from fitness landscapes, allows us to build quantitative null models for the relationship between community structure (i.e., species composition) and function. Combining this theory with experiments, we seek to precisely determine the contribution of interactions of different order (e.g., pairwise, third-order, etc.) and type (e.g., modifying population growth through competition or facilitation, or altering the expression of this function at the cellular level).

## Results

### A simple additive function in a simple in vitro consortium

**Fig 1:**
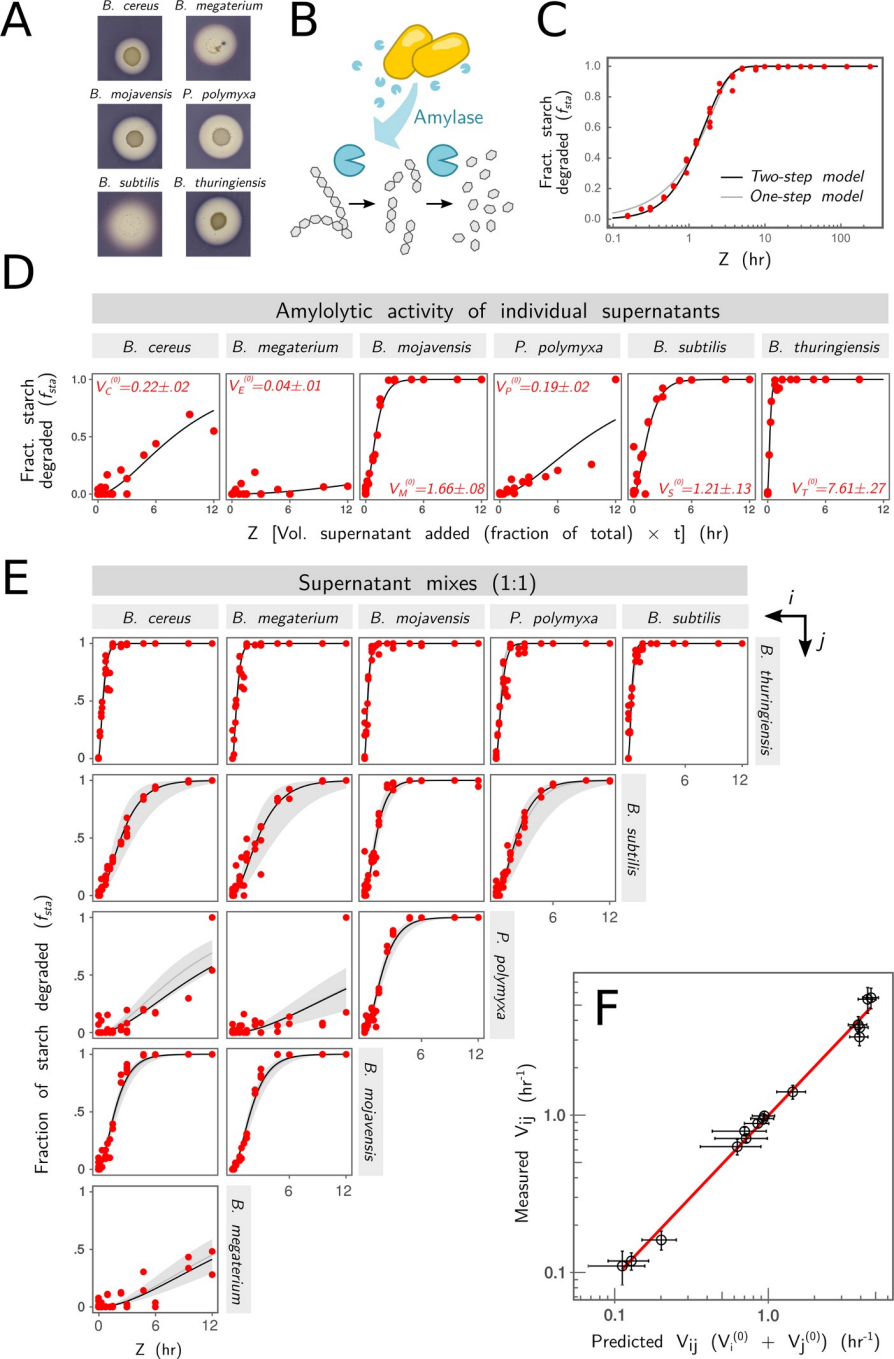
Starch degradation of simple consortia follows an additive null biochemical model. (A-B) All members of our six-species consortium are able to break down extracellular starch by secreting extracellular amylases. This is evidenced by the presence of a halo around colonies of all six species after Lugol stain [52]. Colonies formed on basic growth minimal media (1x bSAM) agarose plates. Extracellular *Bacillus* amylases bind randomly on starch chains, breaking them at random positions [53]. We propose a two-step enzymatic degradation model (Methods) that requires two cleavage reactions to turn starch into smaller oligosaccharides. (C) We fit the model (Eq 9) to the result of incubating purified *B*. *subtilis* amylase at various concentrations (from 0.4 to 100 μg/mL in 2X increments) with a 1 mg/mL starch solution for various lengths of time. In the horizontal axis, we plot *z = x⋅t*, where *x* represents the dilution of the enzyme relative to the maximum concentration (see Methods), and *t* is the incubation time. In the vertical axis, we plot the fraction (“Fract.”) of starch degraded for each condition. Each red dot represents a measurement for a different value of (*x*, *t*). (D) Amylolytic activity of the supernatants of individual species in monoculture. Species were grown in monoculture in 3 mL of 1x bSAM medium at 30°C for 24 hr. The vertical axis represents the fraction of starch degraded by different dilutions (*x =* 0.05, 0.125, 0.25, 0.5) of the filtered supernatants incubated at 30°C with 1 mg/mL starch for various time lengths (t = 3, 6, 19, 24 hr). The two-step model (Eq 9; solid black line) is fit to the data (red dots), and the fitted parameter *V*_*i*_ (hr^−1^), which corresponds to the function of the monocultures *V*_*i*_^*0*^, is reported in red in each subpanel. (E) Amylolytic activity of 1:1 mixtures of all possible pairs of single-species filtered supernatants. In addition to experimental data (red points) and fit to Eq 9 (solid black line; fitting parameter *V*_*ij*_), we also show the fraction of starch degraded for each value of *z* predicted by the null biochemical model of independently acting enzymes (Eq 11; mean ± 2SE, gray region). (F) Comparison of fitted versus predicted values of *V*_*ij*_ for the same pairs shown in (E) (error bars represent ± 2SE); red line represents perfect prediction. Vol., volume.

We sought to study a small synthetic consortium that could be combinatorially reconstituted in vitro and that performs a simple function that could be mechanistically and quantitatively understood from first principles when interactions among species are absent. To this end, we constructed a set of synthetic consortia consisting of every combination of six amylolytic soil bacteria: *Bacillus subtilis*, *B*. *megaterium*, *B*. *mojavensis*, *Paenibacillus polymyxa*, *B*. *thuringiensis*, and *B*. *cereus* (Fig 1A). As the function, we chose the starch hydrolysis rate of the enzymes released by the consortia (Fig 1B and 1C). This function was expressed on agar plates as well as in liquid culture (Fig 1A–1D) by all members of the consortia in isolation. This is a redundantly distributed, simple function that requires just one kind of extracellular enzyme (an endoamylase) for its completion.

To formulate a quantitative null model for this function that is based on the underlying biochemistry, we adopted the random or “single-attack” model of enzymatic starch hydrolysis, which has been previously found to capture the mechanism of *Bacillus* endoamylases [53]. In the Methods section, we explain how we mathematically formulated this model and considered two alternative, minimal scenarios that are consistent with the concentration of low-molecular-weight soluble starch that we use as substrate and the operational definition of starch we use throughout the paper (Methods). As we will show in what follows (Fig 1E and 1F), the model also has a strong predictive power. As described in Methods, enzymatic starch hydrolysis is well captured under our experimental conditions by a sequential two-step Michaelis-Menten reaction with each step having an identical velocity (*V*) (Figs 1B and 1C and S1).

We now sought to apply this model to the extracellular enzymes from live cultures of the species in the consortium. The same two-step model in (Eq 9) also fits well the kinetics of starch degradation by the amylases released into the extracellular medium by each species in monoculture (Fig 1D), and the fitting parameter *V*_i_ gives us the hydrolysis rate of each species *i*. Moreover, as described in the Methods section, the theory of chemical kinetics predicts that the starch hydrolysis rate (*V*_*AB*_) of a cocktail or mixture of the enzymes independently released by monocultures of species A and B is the sum of both rates in isolation (*V*_*AB*_ = *V*_*A*_^(*0*)^ + *V*_*B*_^(*0*)^). We tested this prediction by growing all six species in monoculture for 24 hr and then mixing their supernatants 1:1 in every possible pairwise combination (Fig 1E). A series of different volumes (representing enzyme dilutions *x* = 0.05, 0.125, 0.25, 0.5) of these supernatant mixtures were incubated at T = 30°C with a 1 mg/mL starch solution for various incubation times (3, 6, 19, and 24 hr), and the fraction of starch degraded at each time was then measured for each condition (Methods). A fit to the model in Eq 9 allowed us to obtain the degradation rate of the enzymatic cocktails (*V*_*AB*_), which can be compared to the prediction from the model of independently acting enzymes (Fig 1E). We find a very strong correlation (Pearson’s *ϱ* = 0.973, *P* < 0.001; root-mean-square deviation [RMSD] = 0.41, *N* = 15) between the predictions from this naturally additive, interaction-free mechanistic model and the experimental measurements (Fig 1F). The strong predictive power of the two-step model further validates it under our experimental conditions.

In summary, the theory of chemical kinetics states that the rate of hydrolysis when two or more different enzymes are mixed together should be the sum of the hydrolytic rate of each enzyme, if those enzymes act independently on the substrate. This prediction is validated in our experiments. When the species in our consortia are grown in separate culture tubes and do not interact with one another in any way, their contributions to the rate of starch degradation add up. Armed with this mechanistic null model, we are now prepared to investigate the role of pairwise and higher-order functional interactions.

### Mapping pairwise functional interactions in our microbial consortium

To investigate the contribution of pairwise and higher-order interactions to the function of our consortia, we made use of concepts and tools that were originally developed for the study of genetic interactions. By analogy with the theory of fitness landscapes, we define the functional landscape as a map connecting every possible combinatorially assembled consortia with its function (which, in the case at hand, represents the amylolytic rate of the cocktail of enzymes collectively secreted by a consortium).

**Fig 2:**
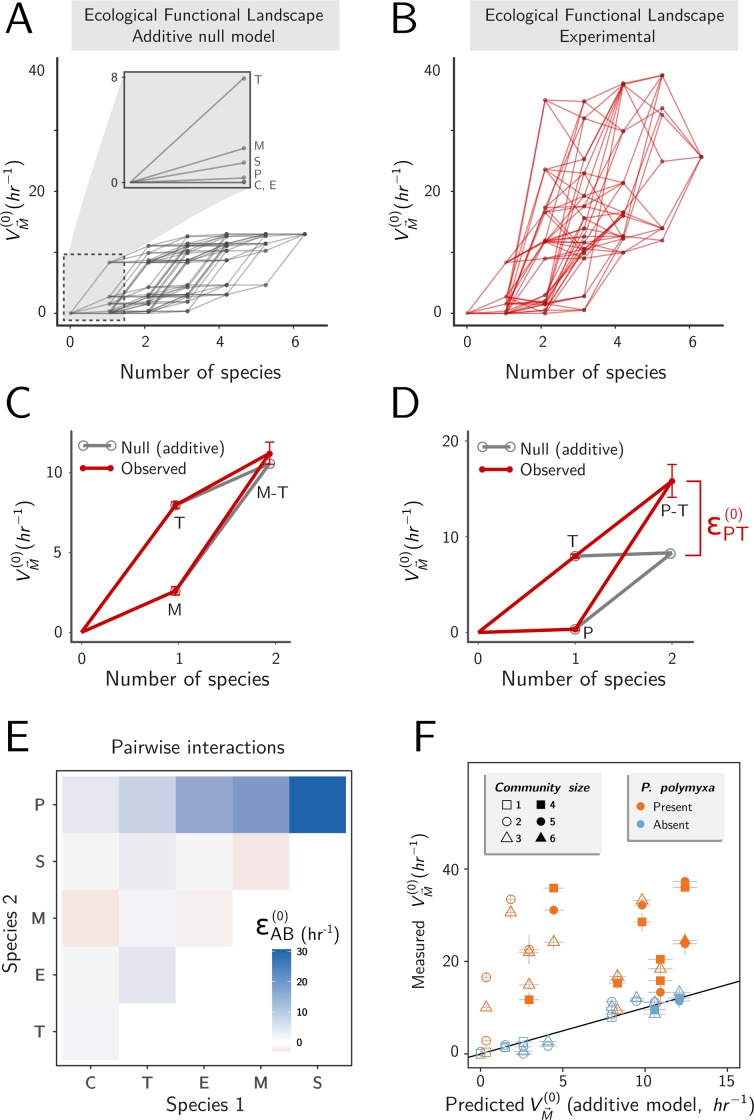
Pairwise interactions are ubiquitous in our microbial consortia. (A) Functional landscape predicted by the additive biochemical model (Eq 11). (B) Experimental functional landscape, where each point is the experimentally measured amylolytic rate of a consortium (error bars omitted for clarity). (C) Example of a pairwise functional landscape that is representative of communities lacking *P. polymyxa* (in this case, *B*. *mojavensis* and *B*. *thuringiensis*). Red lines show experimental measures (±SE), which are well predicted by the additive model (gray). (D) Functional landscape for a pairwise consortium containing *P. polymyxa* and *B*. *thuringiensis*. Here, the experimentally measured function of the pair (red) is not well predicted by the sum of the individual contributions (gray). The difference $\varepsilon_{PT}^{\left(0\right)}$ quantifies the pairwise interaction. (E) Pairwise interactions ($\varepsilon_{AB}^{\left(0\right)}$) of all possible two-member species consortia. (F) Comparison of the measured community function and the function predicted by the additive model (±2SE). Shape and color represent community size and presence of *P. polymyxa*, respectively. Black line represents perfect prediction. Species are designated as follows: C = *B*. *cereus*; E = *B*. *megaterium*; M = *B*. *mojavensis*; P = *P. polymyxa*; S = *B*. *subtilis*; and T = *B*. *thuringiensis*. This convention will be used throughout the text.

In the absence of interactions, growing species together would be identical to growing them separately in their own culture flask. In such scenario, the null mechanistic model described above predicts that the functional landscape should be additive, and the function of a consortium would grow monotonically with the number of species (Fig 2A). For an *M*-species consortium (which we denote as $\vec{M}$), our null model is given by Eq 11 (Methods) and can be written as:

$$V_{\vec{M}}{}^{\left(0\right)}=\overset{M}{\underset{i=1}{\sum }}V_{i}{}^{\left(0\right)},$$

where ${V_{\vec{M}}}^{\left(0\right)}$ denotes the function of the consortium, and *V*_i_^(0)^ represents the amylolytic rate of the enzymes released by species *i* in monoculture.

To test the null model, we assembled combinatorial consortia including pairs and trios as well as four-, five-, and six-member species and measured the amylolytic rates of the enzymes released over 24 hr of culture (Methods). For internal consistency, we also replicated the monocultures, obtaining good agreement with the measurements in Figs 1 and S1B. Overall, the experimental functional landscape often exhibits a marked deviation from the prediction of the null model (Fig 2B), indicating the presence of interactions in many—but not all—consortia. For instance, we show the functional landscape for two different two-species consortia: one formed by *B*. *thuringiensis* (“T”) and *B*. *mojavensis* (“M”) (Fig 2C) and a second one formed by *B*. *thuringiensis* and *P. polymyxa* (“P”) (Fig 2D). As we see in Fig 2C, the null model approximates well the function of the pairwise consortium formed by *B*. *thuringiensis* and *B*. *mojavensis*. In contrast, the null model underestimates the function of the consortium formed by *B*. *thuringiensis* and *P. polymyxa* (Fig 2D). The deviation between the null model prediction and the measured function of the consortium quantifies the pairwise functional interaction, which we denote as *ε*_PT_^(0)^ (i.e., *V*_*PT*_^(0)^ = *V*_*P*_^(0)^ + *V*_*T*_^(0)^ + *ε*_PT_^(0)^) (Fig 2D).

The strength of pairwise functional interactions can be determined in the same manner for other pairs of species too (Fig 2E). A pattern readily emerges: interactions are strong when *P. polymyxa* is present but weak when it is absent. Consistent with this finding, the predicted function from the additive null model is strongly predictive of the measured function (RMSD = 1.47, *N* = 25) for all of the consortia we tested (including all of those with *M* > 2 species), where *P. polymyxa* is absent (Fig 2F, blue dots). On the other hand, when *P. polymyxa* is present, the additive null model is weakly predictive (Fig 2F, RMSD = 17.36, *N* = 28), severely underestimating the amylolytic function of all consortia. Given the failure of the null model, we conclude that pairwise and potentially also higher-order functional interactions are required to explain the function of consortia when *P. polymyxa* is present.

### Quantifying high-order functional interactions in our simple consortia

**Fig 3:**
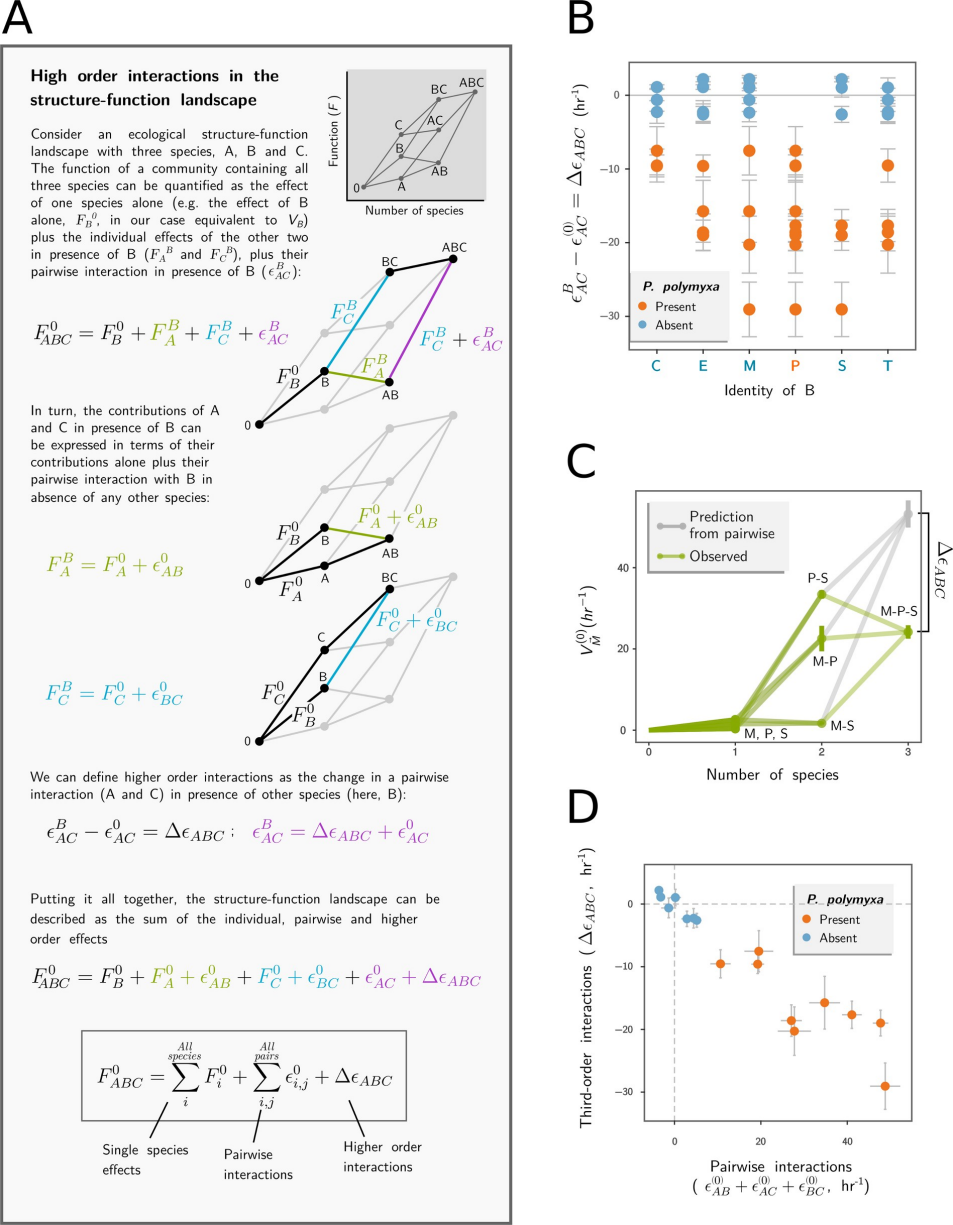
Higher-order interactions in simple amylolytic consortia. (A) Box explaining how single, pairwise, and higher-order interactions in the function of microbial consortia can be separated and quantified using ecological functional landscapes. (B) Third-order interactions *Δε*_*ABC*_(±SE), defined as the difference in the functional interaction ($\varepsilon_{AC}^{\left(0\right)}$) when a pair “AC” is grown alone and in the presence of a third species “B” ($\varepsilon_{AC}^{\left(B\right)}$), whose identity is shown in the horizontal axis. Species are designated as C, *B*. *cereus*; E, *B*. *megaterium*; M, *B*. *mojavensis*; P, *P. polymyxa*; S, *B*. *subtilis*; and T, *B*. *thuringiensis*. (C) Community function *V* (±SE) for every combinatorial consortia of *P. polymyxa* (“P”), *B*. *mojavensis* (“M”), and *B*. *subtilis* (“S”). In gray, we show the function predicted by adding the functions of all three taxa in isolation and all pairwise interactions (rather than, for instance, by averaging out the pairwise interactions). (D) Third-order interactions (±SE) are strongly anticorrelated (Pearson’s *ρ* = −0.95, *P* < 0.001) with the sum of all pairwise interactions (±SE in hr^−1^).

Third-order interactions capture how the function of a pair of species (e.g., the amylolytic rate of the enzymes secreted by the pair) is altered when a third species is present. To determine whether pairwise interactions can indeed be altered by the presence of a third species, we measured the amylolytic rate for every possible pairwise and three-member consortia and then followed the approach outlined in Fig 3A. The results are summarized in Fig 3B, and they show that a model that adds up all single-species contributions and all pairwise interactions vastly overestimates the amylolytic function of our consortia when *P. polymyxa* is present. An example is given in Fig 3C, which shows a three-member community formed by *P. polymyxa* (“P”), *B*. *subtilis* (“S”), and *B*. *mojavensis* (“M”). None of the three species have a strong amylolytic activity in monoculture (Fig 1D), but the two pairs that include *P. polymyxa* have high amylolytic rates, driven by strong pairwise interactions (Fig 3C). However, as shown in Fig 3C, the trio has much lower function (*V*_*PSM*_^0^ = 24.2 ± 1.6 hr^−1^) than would be expected by adding together the single-species contributions and every possible pairwise interaction (53.2 ± 3.3 hr^−1^). The same finding extends to other trios, and we find that third-order interactions generally exhibit a strong negative correlation with the sum of all pairwise interactions (Pearson’s *ϱ* = −0.95, *P* < 10^−10^; Fig 3D). By comparison, the correlation with first-order interactions is much weaker (*ϱ* = −0.29, *P* = 0.04; S2 Fig), suggesting that pairwise interactions do not add up as one may predict based on the functional landscape model in Fig 3A. Rather, the result in Fig 3D shows that pairwise interactions combine subadditively.

In principle, interactions that affect the function of a consortium could be caused by any combination of (1) synergy or antagonism between the contributions of individual members to the net function of the consortium; (2) ecological competition (facilitation), which decreases (increases) the population size of community members relative to their monocultures (potentially altering the amount of function contributed by each population); or (3) an increase or decrease in the per-cell contribution to the net function of the consortium when other species are present (S3 Fig). The success of our null biochemical model for all enzymes rules out the possibility of (1) biochemical synergy involving the enzymes released by *P. polymyxa*. In the following sections, we will proceed to examine whether (2) facilitation or (3) stimulation of amylase expression could explain the higher-than-expected function of consortia containing *P. polymyxa*.

### Facilitation qualitatively explains the enhanced function of consortia containing *P. polymyxa*

**Fig 4:**
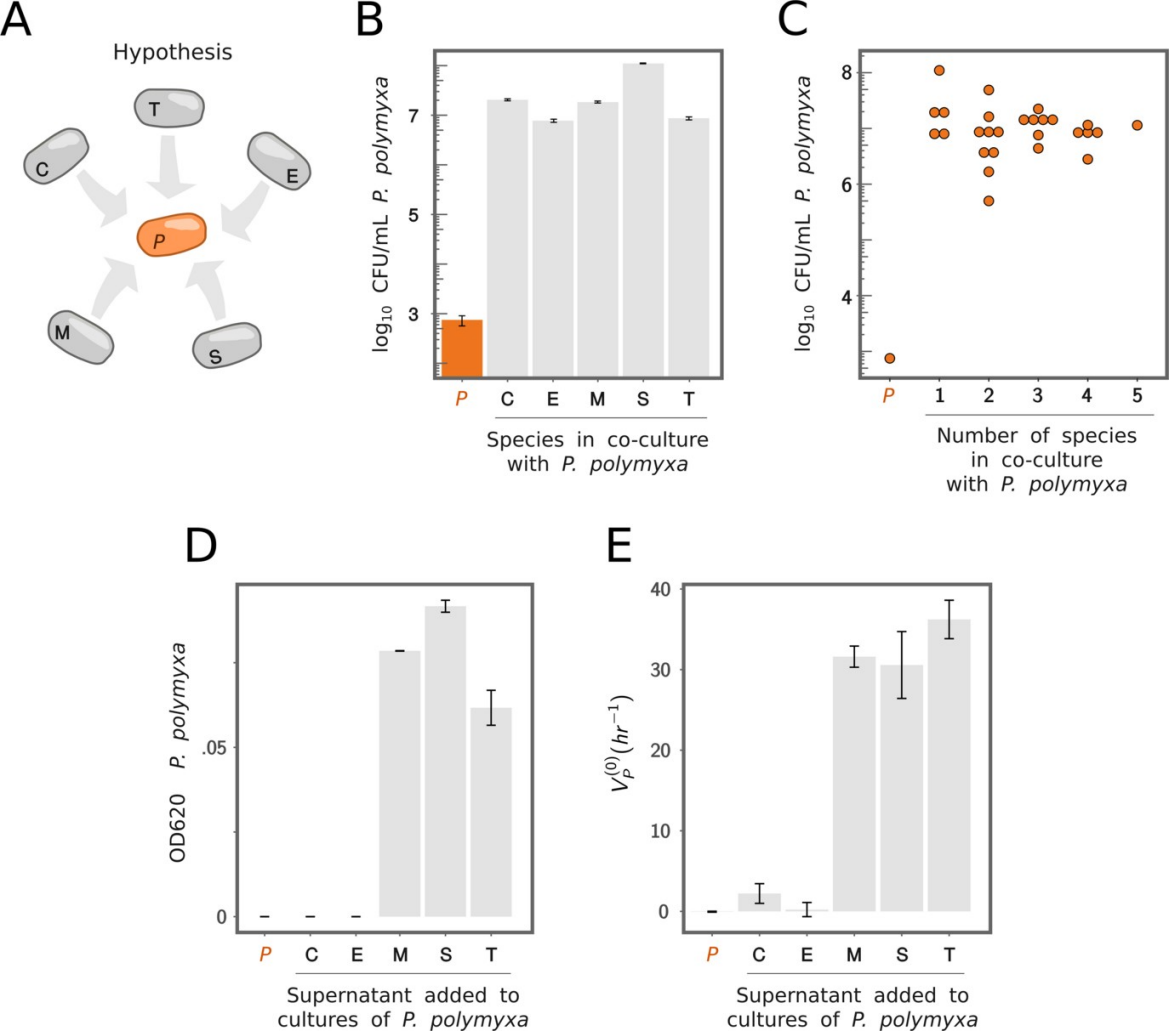
Redundancy in the ecological facilitation of *P. polymyxa* explains higher-order interactions. (A) If growth of *P. polymyxa* is facilitated by any and all of the other species, this facilitation may be redundant. Species are designated as C, *B*. *cereus*; E, *B*. *megaterium*; M; *B*. *mojavensis*; P, *P. polymyxa*; S, *B*. *subtilis*; and T, *B*. *thuringiensis*. (B) *P. polymyxa* grows in the presence of any of the other species (gray bars) but not in monoculture (orange bar). CFUs were determined by colony counting of serially diluted cultures after 48 hr of growth at 30°C. Error bars represent ±SE. (C) *P. polymyxa* grows to a comparable density as a part of a pair, trios, or higher numbers of other species in the consortium. This strongly suggests redundancy in the facilitation mechanism. (D) *P. polymyxa* growth (quantified by the final OD_620_ after 24 hr of culture, initialized at OD_620_ = 0; mean ± difference of two independent biological replicates) in our growth media supplemented with a 1:10 dilution of the filtered supernatant of each of the other species (Methods). (E) Function (*V*_*P*_^*(0)*^) of *P. polymyxa* monocultures shown in (D) (mean ± difference of two independent biological replicates). CFU, colony forming unit; OD, optical density at 620 nm.

Given that *P. polymyxa* grows poorly in our medium, we hypothesize that the positive pairwise interactions between every other community member and *P. polymyxa* may arise as a result of facilitation toward *P. polymyxa* (Fig 4A). To test this hypothesis, we determined the number of colony forming units (CFUs) of *P. polymyxa* in monoculture as well as in coculture with each of the other members of the consortium. Consistent with our hypothesis, we find that *P. polymyxa* is facilitated by every other member of the consortium (Fig 4B), but their facilitative effects are redundant: coculturing *P. polymyxa* with two or more members of the consortium produces a similar growth stimulation than that observed in coculture with just one other member (Fig 4C).

Growth stimulation is also observed when *P. polymyxa* is grown in media supplemented with the filtered supernatant of every other species in monoculture (with the exception of *B*. *megaterium* and *B*. *cereus*, Fig 4D). This ecological facilitation also results in a marked increase in the amylolytic activity of *P. polymyxa* (Fig 4E). To ensure that this increase in amylolytic activity is not due to the activity of extracellular amylases carried over in the *Bacillus* spent media, we filtered these a second time through a 30-KDa filter membrane (Methods), which blocks the passage of amylases (molecular weight [MW] > 50 KDa). The filtrate through the second 30-KDa filter exhibited no amylolytic activity (S4 Fig), demonstrating that amylases are not present in it. Despite the lack of growth stimulation by the supernatants of *B*. *megaterium* and *B*. *cereus* monocultures, growth of *P. polymyxa* is strongly facilitated by either of them (Fig 4B).

Altogether, these results suggest that cross-feeding facilitation stimulates the growth of the *P. polymyxa* population, which in turn leads to a larger amount of amylase secreted (Fig 4E). We also find that all of the *Bacillus* species in our consortia are able to facilitate the growth of *P. polymyxa* in pairwise coculture with it, supporting our hypothesis that this facilitation is redundant: any combination of the *Bacillus* species stimulates the growth of *P. polymyxa* about as much as any of these species do separately (Figs 4C and S5). This is overall consistent with the finding reported in Fig 3C, that the positive effect of adding additional species to a *P. polymyxa* consortium qualitatively saturates after the first partner is added. These results prompt the question of whether ecological facilitation alone is sufficient to quantitatively explain the observed interactions in our consortia. If that were the case, then by including population growth into our null model, we could then predict the amylolytic function of *P. polymyxa* consortia.

### Including population dynamics into a null model of the structure-function landscape

To address this question, we follow the same strategy we used before and formulate a new additive null model that explicitly incorporates population growth while assuming that no other interactions exist (S3 Fig). Any systematic deviations from this new null model will reveal additional interactions—for instance, those that modulate the amount of amylase expressed per cell. To formulate this new null model, we must determine how the amount of amylase produced by a species (which is proportional to *V*_i_) depends on how much its population (*N*_i_) grows over the incubation time *T* (i.e., *ΔN*_i_ = *N*_i_(*T*) − *N*_i_(*0*)). In our experiments *N*_i_(*T*) >> *N*_i_(*0*), so the population growth is approximately equal to the population size at the time of harvest (*ΔN*_i_ ≈ *N*_i_(*T*), which for simplicity of notation we denote as *n*_i_). Once we have determined how *V*_i_ depends on *n*_i_ for each of the *M* species (which we will hereafter denote as *V*_i_(*n*_i_)), our additive null model now incorporates population growth and takes the form:

$$V_{\vec{M}}{}^{\left(0\right)}=\overset{M}{\underset{i=1}{\sum }}V_{i}\left(n_{i}\right).$$

The key is then to determine the functions *V*_i_(*n*_i_). As a first ansatz, we considered a simple model in which the amylolytic function of each species is assumed to be proportional to its population size at the time of harvest, i.e.,

$$V_{i}\left(n_{i}\right)=\lambda_{i}n_{i},$$

where *λ*_*i*_ reflects the average amount of amylase produced per cell (Methods). Beyond being the simplest [54] (Holling type I) functional response, the proportionality between amylolytic function and population growth arises naturally in a minimal mathematical model that assumes constitutive expression of the amylase gene and exponential population growth (Methods). Given this first ansatz, our new null model for the function of a consortium would take the form ${V_{\vec{M}}}^{\left(0\right)}=\sum_{i}\lambda_{i}n_{i}$. To construct this model, we reexamined all of the mixed-species consortia in Fig 2 and determined *n*_i_ for all species by plating methods (Methods). To estimate *λ*_*i*_, we simply divided the amylolytic rate of each monoculture by the final number of cells *n*_i_ at the time of harvest (T = 24 hr; Methods).

**Fig 5:**
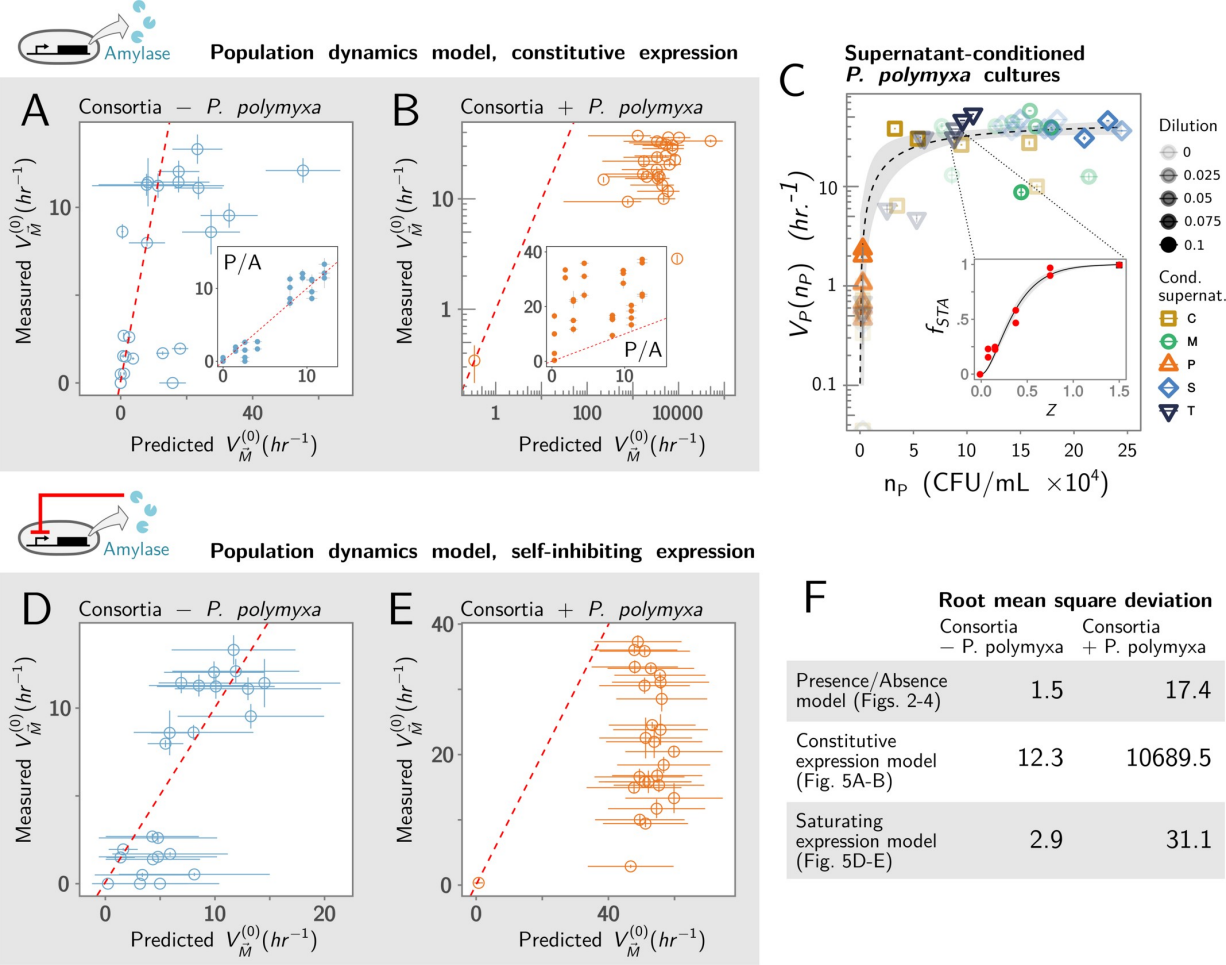
Including population dynamics into a null model of the structure-function landscape. (A-B) We compare the predictive ability of the P/A null model (insets; data replotted from Fig 3F), with the revised null model that assumes that species contribution to function is proportional to their population size. For clarity, we separate consortia where *P. polymyxa* is absent (A) from those where it is present (B). Note the log-log scale used here for easier interpretation of the data. Red dashed line represents the identity curve where the null model perfectly predicts the function of the consortia. (C) To test whether the relation between final population size and function in *P. polymyxa* is well approximated by a saturating function, we grew *P. polymyxa* in media supplemented with different amounts of spent media from each of the other taxa. We determined the final population size in CFU/mL and the amylolytic function *V* (Methods, example in inset). The data were fitted using the saturating function $V_{\vec{M}}^{\left(0\right)}=\sum_{i=1}^{M}u_{i}\frac{n_{i}}{K_{i}+n_{i}}$ (dashed line); 95% confidence interval (gray shading). (D) Comparison of the measured community function and the function predicted by the model assuming that function saturates with population size, for consortia where *P. polymyxa* is absent, and (E) for consortia in which *P. polymyxa* is present. All points shown ±SE. In (A), (B), (D), and (E), red dashed line represents perfect prediction. CFU, colony forming unit; Cond. supernat., Conditioning species supernatant; P/A, presence/absence.

The population growth for all species is generally different in mixed culture than in monoculture (S5 Fig). Yet, the null "presence/absence" (P/A) model that we introduced in Fig 2 (which ignored the effect of population growth) predicts function better than the revised null model (which assumes that amylolytic activity is proportional to the population size). This is true regardless of whether *P. polymyxa* is absent (Fig 5A, RMSD = 12.34 versus 1.47 [inset]; *N* = 25) or present (Fig 5B, RMSD = 10,689.5 versus 17.36 [inset]; *N* = 28). We reasoned that this could be explained if the amount of amylase produced by a population saturates and becomes insensitive to population size past a certain population threshold. To capture this hypothesis, we propose a new ansatz in which the amount of amylase produced by species *i* over an incubation time *T* (and thus the enzymatic rate *V*_i_) is a generic Holling type II saturating function of its final population size:

$$V_{i}\left(n_{i}\right)=u_{i}\frac{n_{i}}{n_{i}+K_{i}}.$$

Here, *K*_*i*_ represents the threshold population size for species *i*, and *u*_i_ represents the maximum amylolytic rate of species *i*, which reflects the amount of amylase secreted when *n*_*i*_ is much higher than *K*_*i*_. As we show in the Methods section, a minimal generic model of amylase expression under negative-feedback regulation naturally exhibits a saturating dependence between *V*_*i*_ and *n*_i_ (S6 Fig). Encouragingly, amylase expression in multiple *Bacillus* species (as well as in *P. polymyxa*) [55,56] is under negative-feedback regulation, inhibited through catabolite repression by the byproducts of its enzymatic action over starch. A similar form of a feedback loop has also been reported for other extracellular enzymes [39,57].

To test the validity of this ansatz, we measured the growth and amylolytic activity of *P. polymyxa* monocultures supplemented by different dilutions (ranging from 1:10 to 1:40) of spent media from each *Bacillus* species in monoculture (Methods). Projecting the measured *V*_P_ and *n*_P_ for all of these experiments on the same plot, we find that, to a good approximation, the amount of amylase produced by *P. polymyxa* in monoculture is well predicted by its final population size and it is well fitted by the generic “type II” saturating function introduced above (Fig 5C; Methods). Encouraged by this result, we repeated the experiment for all other members of the consortia to see whether their function also saturates with population growth (S7 Fig). The results were again consistent with the saturating model. By fitting the type II Holling function to the data (Methods; S7 Fig), we estimated the asymptotic amylolytic function *u*_i_ and the threshold population size *K*_i_ for each species. Armed with these fits, we propose a new additive null model for the function of an *M*-species consortium:

$$V_{\vec{M}}{}^{\left(0\right)}=\overset{M}{\underset{i=1}{\sum }}u_{i}\frac{n_{i}}{n_{i}+K_{i}}.$$

This model assumes, as before, that the enzymes released by each species act additively on the starch substrate and that the amount of amylase secreted by each species is deterministically governed by the number of cells in the population. Interactions exclusively affecting population dynamics will increase or decrease *n*_i_ (but not *u*_i_ or *K*_i_), so their effect on the overall function is implicitly captured by the null model above, if no other interactions exist.

### Saturating effects of population growth explain the success of the original P/A null model

The observed insensitivity of amylolytic function to competitive interactions among the *Bacillus* strains could be explained if their population sizes were generally higher than the threshold *K*_*i*_. To test whether this is true, we compared the final population size of all species with their respective population thresholds *K*_i_. We found that in virtually all cases, *n*_i_ > *K*_i_ (S8 and S9 Figs). This means that, at the population densities reached by the members of our consortia, the contribution of each species to the total amylase pool becomes insensitive to the final population size. In this limit, we can approximate our revised null model as ${V_{\vec{M}}}^{\left(0\right)}\approx \Sigma_{i}u_{i}$. For consortia lacking *P. polymyxa* *u_i_* ≈ *V_i_*^(0)^ (S9 Fig), so the revised null model that includes the effect of population size converges to our initial P/A null model (${V_{\vec{M}}}^{\left(0\right)}=\Sigma_{i}{V_{i}}^{\left(0\right)})$ (S10 Fig, Pearson *ϱ* = 0.88, *P* < 10^−7^, RMSD = 2.21; *N* = 25). This explains why our initial P/A null model, which ignored population growth, predicts so well the amylolytic function of microbial consortia despite the strong competitive interactions among them (S5 and S9 Figs).

Using the values of *u*_i_ and *K*_*i*_ estimated from the fits in Figs 5C and S7, we calculate the predicted function for our multispecies consortia using the new null model (Fig 5D and 5E). As before, the null model is strongly predictive of function in consortia that lack *P. polymyxa* (RMSD = 2.98, Fig 5D). Yet, it still systematically overestimates the function of those that include it (RMSD = 31.1, Fig 5E and 5F). This suggests that interactions between *P. polymyxa* and the other members of the consortia do more than simply altering the population growth of *P. polymyxa*: they also generally lower the per-cell production of amylase (Methods).

### Redefining pairwise and HOIs from an additive null model that includes population dynamics

We started this section by asking whether the higher-than-expected function of consortia containing *P. polymyxa* is caused by facilitation (a population dynamics interaction) or stimulation of amylase expression (a behavioral interaction). Can we quantitatively separate the effect of both types of interactions? In the Methods section, we mathematically demonstrate that the pairwise interactions ($\varepsilon_{AB}^{\left(0\right)}$) that we had determined in Fig 2E as deviations from the original P/A null model can be partitioned as the sum of two separate terms. One of these terms captures the mutual effect that both species have on their respective population dynamics ($\varepsilon_{AB}^{pop}$). The second term quantifies the change of amylase expression in coculture relative to monoculture (i.e., a behavioral interaction term $\varepsilon_{AB}^{behav}$) (Methods; S11 Fig).

**Fig 6:**
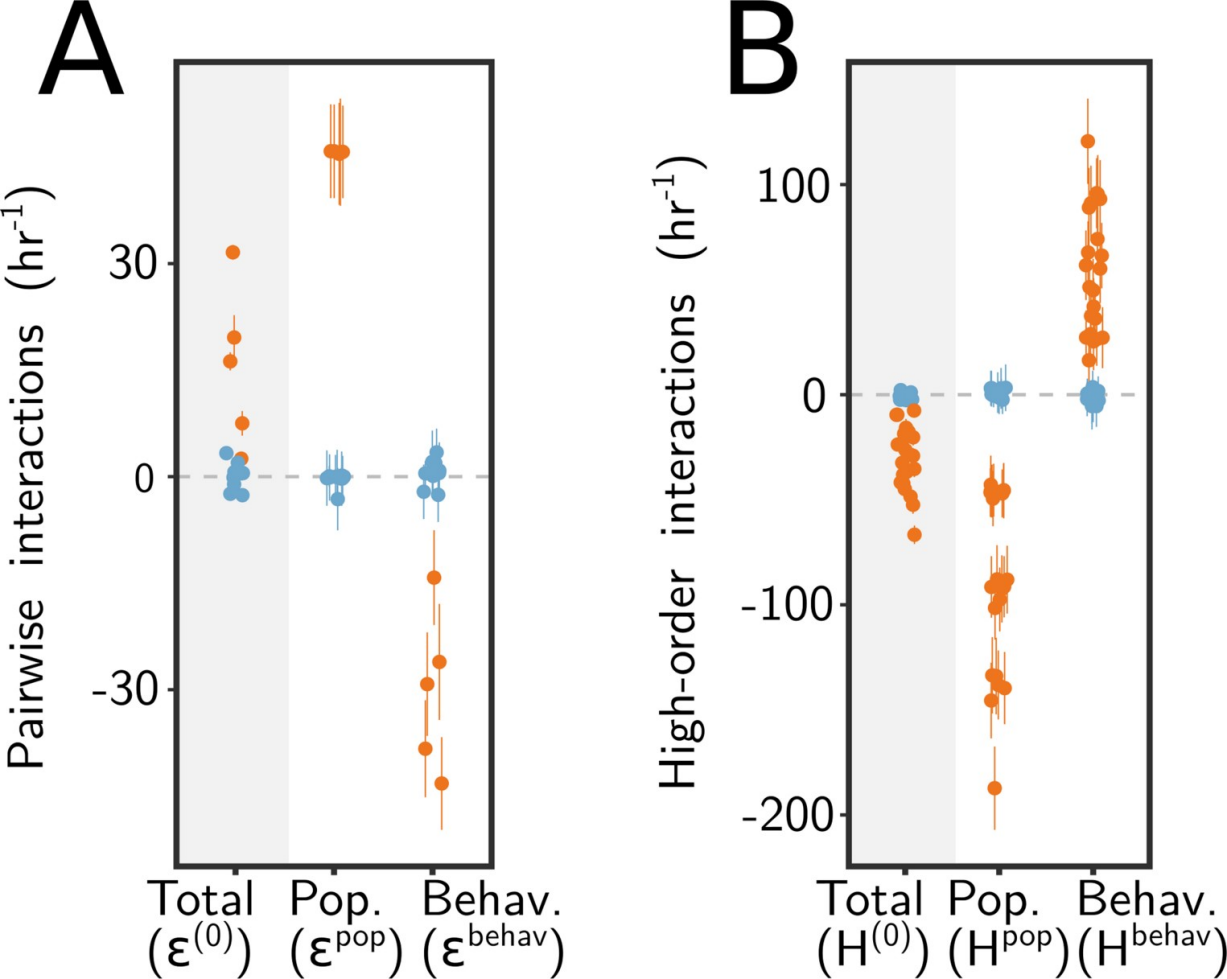
Quantifying the population (“Pop.”) dynamics and behavioral (“Behav.”) components of both pairwise and high-order interactions. (A) Pairwise interactions (±SE) for all two-species consortia. We separate pairs by whether they include (orange) or do not include (blue) *P. polymyxa*. (B) Higher-order interactions in communities with three or more members. Similar to (A), we differentially color consortia by whether they include (orange) or lack (blue) *P. polymyxa*. Jitter in the horizontal axis (which is categorical) was added to the data for clarity of presentation.

We applied this analysis to our pairwise communities containing *P. polymyxa* (Fig 6A). We find that facilitation interactions are higher than the net interaction and that the effect of facilitation is partially compensated by inhibition (rather than stimulation) of amylase expression in coculture. Our experimental methods do not allow us to distinguish the amylases contributed from each species in isolation. However, using the fact that amylolytic rates cannot be negative, we can unambiguously determine that *P. polymyxa* expresses less amylase in coculture than it does in monoculture. A quantitative estimation of the upper and lower bound of the inhibition of amylase production by *P. polymyxa* in mixed culture is shown in S11 Fig.

A similar logic was applied to the net effect of HOIs. We show in the Methods section that these also can be partitioned as the sum of population dynamics interactions and behavioral interactions. We quantified both contributions (Methods) and plot them in Fig 6B. We find that both behavioral and population dynamics interactions contribute to the HOIs, and their magnitudes are similar though of different sign. We conclude that accounting for population dynamics into the null model does not eliminate the effect of HOIs in distorting the functional landscape. We then asked whether a similar compensation could be masking the presence of population dynamics or behavioral interactions in consortia lacking *P. polymyxa*. As shown in Fig 6A and 6B, and consistent with our previous conclusions, we find that neither population dynamics nor behavioral interactions are significant for species other than *P. polymyxa*.

## Discussion

The results presented above represent an attempt to quantitatively map the functional landscape of a microbial consortium. All of our results hinge on the definition of functional interactions as deviations from the predictions of a null model of the structure-function landscape. Functional interactions are observed when the contribution of a community member (or set of members) to a community-level function depends on the presence or absence of other species. For instance, pairwise interactions reflect how the contribution of a single species to a community function (e.g., in the case explored above, the amylolytic activity of the enzymes it secretes) depends on whether it is grown alone or in coculture with a second species. This may be mediated by behavioral interactions, in which microbes alter the phenotype of neighbors when grown in coculture [39,58,59] (S3 and S11 Figs), or by population dynamics interactions, in which a population of microbes influence the population size of a second species [58,60] (S3 and S11 Figs), or by both [58,61]. In the case studied here, amylase expression and secretion is often tied to cell growth in *Bacillus* sp. [62,63], which indicates that both ecological and behavioral interactions are not necessarily independent of one another.

By analogy with pairwise interactions, third-order interactions capture how the function of a pair of species, (e.g., the amylolytic rate of the enzymes secreted by the pair) is altered when a third species is present. This simple idea, based on the study of fitness landscapes and complex interactions in genetics [41,64,65], allowed us to decompose the function of a community into the contributions of single species and the interactions that modulate these contributions and can be used to shed light onto the role played by HOIs in community function. As we have shown here and others have shown before in different contexts [41], a null model of how the functional contributions of multiple species should combine to determine the community function is essential to unequivocally identify interactions through this approach.

We started this paper by showing that an additive null model predicts the amylolytic rate of our consortia when no interactions are present. Our experiments revealed that the additive null model has an unexpectedly strong predictive power when any combination of the *Bacillus* species is cocultured together. The strength of our additive null model may thus reflect that no interactions are present. Yet the population size of *Bacillus* species in mixed consortia generally reaches different levels than in monoculture, indicating that population dynamics interactions are indeed present and they can be strong.

By contrast, when *P. polymyxa* is part of the consortia, the additive model failed, indicating the presence of interactions. We found strong evidence of facilitation from the *Bacillus* species toward *P. polymyxa*, and this prompted us to ask whether we could explain these interactions by including the population size of *P. polymyxa* in an updated null model. We first assumed a linear correspondence between population size and the amount of enzyme produced. This actually diminished the predictive power of our null model for all consortia. To explain this seemingly paradoxical result, we measured the precise dependence between population size and amylolytic rate in monoculture and found that it is not linear. Rather, the amylolytic activity has a saturating relationship with population size, becoming insensitive to it above a population threshold. All of the *Bacillus* strains grow above this threshold both in monoculture and in mixed culture. This explains why species interactions that alter population size do not have an effect on amylase production, and additivity is observed when *P. polymyxa* is absent.

In the case of *P. polymyxa* consortia, we asked whether the increase in function observed in mixed culture stemmed from facilitation, from stimulation of amylase production, or from both. Our results identified an important effect of metabolic facilitation. Unexpectedly, however, *P. polymyxa* secretes less enzyme per cell than we would expect based on the monocultures (Figs 6 and S11). Although identifying the molecular causes behind this down-regulation is beyond the scope of this paper, we might speculate that the presence of amylases secreted by other species could result in a stronger catabolite repression (i.e., more inhibition at lower cell densities) compared to the monoculture, leading to the detection of lower per-capita amylase secretion in mixed cultures.

As for the facilitation, the strain of *P. polymyxa* we included in our consortia is a biotin auxotroph [66], a trait that is widely shared with other strains from the same species [67]. Indeed, it grows poorly in our growth medium (which does not include biotin), but its growth is rescued as expected by adding a biotin-containing vitamin supplement (S12 Fig). This suggests that the cross-feeding of biotin (or a biosynthetic precursor of this vitamin) likely mediates facilitation. Despite its importance, facilitation alone was not enough to explain the function of our *P. polymyxa*-containing consortia. The effect of facilitative and of behavioral interactions could be disentangled by formulating a new null model that incorporated the effect of population growth in amylase expression. By disentangling both forms of interactions, we have shown that both can be important contributors to the function of our consortia, particularly when *P. polymyxa* is part of it (Fig 6A and 6B).

The operational detection of interactions as deviations from the prediction of an interaction-free null model has a long history and has been previously used in ecology (e.g., Billick and Case [68]) as well as in many other fields of biology (e.g., [50,51,69–71]). In the context of communities, recent work has used the same analogy with fitness landscapes to detect high-order and complex interactions in ecological systems [6,72]. Our results also emphasize the potential importance of complex functional interactions even for a decidedly noncomplex function: one that could be independently carried out by each species in isolation and that does not require more than a single gene in each species. By comparison to an additive null model, HOIs have also been reported in a set of recent experiments in which the microbiome of *Drosophila melanogaster* was combinatorially reconstituted to map its effects on several life-history traits of the fly host, such as its longevity or developmental time [6].

The confluence of findings between this study and ours suggests that complex functional interactions may play an important role in microbial communities, presenting a fundamental challenge to predicting their function from the bottom up. Indeed, our results suggest that in the presence of higher-order interactions, the ability of our null model to predict community function declines as more species are added to the consortia (S13 Fig). Although this may be seen as a disappointment, it is not unusual in ecology, nor in complex systems in general, that the whole is different from the sum of its parts. Understanding exactly how this complexity works and how the parts come together to produce complex quantitative traits has led to fruitful research in many fields, from genetics and evolutionary biology [73–76] to metabolism [65,77]. We hope that our findings will help stimulate similar efforts in the functional characterization of microbial communities.

## Methods

### Strains and media

Bacterial strains were obtained from ATCC (Manassas, VA, USA) with the following designations: *B*. *subtilis* (ATCC 23857), *B*. *megaterium* (ATCC 14581), *B*. *mojavensis* (ATCC 51516), *P. polymyxa* (ATCC 842), and *B*. *thuringiensis* (ATCC 10792). *B*. *cereus* was isolated from a soil sample in Boston, MA, USA, and identified by 16S Sanger sequencing. To select these species, we screened a larger number of 20 strains of soil bacteria (all from ATCC) that had been reported to be amylolytic. We chose two criteria to include a strain in the consortium: (1) that they must be able to grow in our medium; (2) that they form a halo on agarose plates supplemented with our growth medium after exposure to iodine (indicating that they do secrete amylases under our growth conditions). We discarded *B*. *circulans*, *B*. *clausii*, *B*. *firmus*, *B*. *halodurans*, *B*. *lentus*, *B*. *pumilus*, and *P*. *alvei* for their failure in one or the other of these criteria. We also discarded *B*. *amyloliquefaciens* because its amylolytic activity was so strong that it masked the effects of all other species making their effects undetectable in practice. We discarded *B*. *niacini*, *B*. *simplex*, *Cellulomonas biazotea*, *B*. *mycoides*, and *B*. *licheniformis* for reasons related to their colony morphology and their growth on plates, which made it difficult to work with them. Cell stocks were prepared according to manufacturer instructions and stored at −80°C in 40% glycerol. Basic growth minimal media (1× bSAM) was prepared from 10x concentrated stocks of bM9 Salts containing Na_2_HPO_4_×2H_2_O (85.4 g/L; Bioworld), KH_2_PO_4_ (30 g/L; Fisher Scientific), NaCl (5 g/L; Sigma-Aldrich), NH_4_Cl (10 g/L; Fisher Scientific), supplemented with 0.04% synthetic complete amino acids (w/v; Sunrise Science Products), 0.1% starch (w/v; soluble, Sigma-Aldrich), 1% trace mineral supplement (v/v; ATCC MD-TMS containing EDTA, 0.5 g/L; MgSO_4_∙7H_2_O, 3.0 g/L; MnSO_4_∙H_2_O, 0.5 g/L; NaCl, 1.0 g/L; FeSO_4_∙7H_2_O, 0.1 g/L; Co[NO_3_]_2_∙6H_2_O, 0.1 g/L; CaCl_2_ [anhydrous], 0.1 g/L; ZnSO_4_∙7H_2_O, 0.1 g/L; CuSO_4_∙5H_2_O, 0.010 g/L; AlK[SO_4_]_2_ [anhydrous], 0.010 g/L; H_3_BO_3_, 0.010 g/L; Na_2_MoO_4_∙2H_2_O, 0.010 g/L; Na_2_SeO_3_ [anhydrous], 0.001 g/L; Na_2_WO_4_∙2H_2_O, 0.010 g/L; NiCl_2_∙6H_2_O, 0.020 g/L), CaCl_2_ (0.1 mM; Sigma-Aldrich), and MgSO_4_ (2 mM; Fisher Scientific). Starch assay media (2x SAM) consisted of 2x bM9 salts, supplemented with 0.1% starch (w/v), CaCl_2_ (0.2 mM), and MgSO_4_ (4 mM). To confirm the previously reported biotin auxotrophy of our strain of *P. polymyxa* [66], we supplemented our medium with 1% vitamin supplement (v/v; ATCC MD-VS, which contains folic acid 2.0 mg/L; pyridoxine hydrochloride 10.0 mg/L; riboflavin 5.0 mg/L; biotin 2.0 mg/L; thiamine 5.0 mg/L; nicotinic acid 5.0 mg/L; calcium pantothenate 5.0 mg/L; vitamin B12 0.1 mg/L; p-aminobenzoic acid 5.0 mg/L; thioctic acid 5.0 mg/L; monopotassium phosphate 900.0 mg/L).

### Culture inoculation and combinatorial assembly

Strains were streaked out on BE Starch Agar (0.3% beef extract, 1% starch) plates and grown for 24 hr at 30°C. Seed cultures were started from several colonies (depending on colony size), inoculated into 3 mL 1x bSAM and grown without shaking at 30°C for 24 hr. Cultures were then transferred to a 96-well plate (Corning Cat. No. 3596), and the optical density (620 nm) of 100 μL was measured (Multiskan Spectrophotometer; Fisher Scientific). Cells were harvested by centrifugation at 3,500 rpm for 15 min, washed twice with 1x phosphate buffered saline, and suspended in fresh 1x bSAM media at a concentration of 5 × 10^5^ CFU/μL. Monocultures or combinatorially assembled communities of the six bacilli species were prepared by inoculating 2 μL from each seed culture into 96-deep-well plates (VWR) containing 500 μL of 1x bSAM, regardless of the number of species. The initial density of cells in the six-member consortia was therefore six times higher than in monocultures. Plates were covered with Aerogel film (VWR) and incubated without shaking at 30°C for another 24 hr. Optical density (620 nm) of the grown cultures was measured as above at the end of the new incubation period.

### Determination of amylolytic rates

Starch hydrolysis assays followed the quantitative Lugol iodine staining method described in [78], which is an adaptation of the classic Fuwa method [52]. Lugol staining solution was prepared with 390 mL water: 60 mL Lugol iodine stain (Sigma-Aldrich). Supernatants containing extracellular amylases were prepared for enzymatic assays by applying 330 μL of homogeneously suspended bacterial cultures grown for 24 hr directly to a 96-well 0.2-μm Acroprep filter plate (Pall, Cat. No. 8019) fitted to a 96-well collection plate (Corning, Cat. No. 3596) with a metal collar adaptor (Pall, Cat. No. 5225) and centrifuged for 20 min at 1,500*g* in a tabletop centrifuge (Eppendorf 5810).

Assays, in 96-well plates, contained varying volumes of filtered supernatant (100, 50, 25, 10, and 5 μL), 100 μL 2x SAM, and water to a total reaction volume of 200 μL. Control reaction plates were also prepared for either no enzyme (100 μL 2x SAM and 100 μL water) or no starch with supernatant (100 μL water and 100 μL supernatant). Reactions, assembled with prewarmed SAM, were incubated at 30°C to the desired time point and quenched by transferring 50 μL to a solution containing Lugol iodine stain (130 μL water and 20 μL Lugol per well). The resulting solution was homogenized by pipetting and immediately transferred to a plate reader to quantify starch concentration by measuring the optical density at 690 nm. The amount of starch remaining in the medium is calculated as described in [78], as *f*_*sta*_ = 1 − (*OD*_*690*_(*t*)/*OD*_*690*_(0)). All reactions with no detectable starch degradation were set to *f*_*sta*_ = 0.

### Michaelis-Menten kinetic model of enzymatic starch hydrolysis

We adopt an operational definition of starch as a polysaccharide long enough to give a positive signal in a colorimetric iodine test [52]. To model how the fraction of starch remaining in solution (*f*_*sta*_) depends on the time of incubation *t* and the enzyme concentration [*E*], we examine two simple kinetic models of enzymatic starch hydrolysis. First, we assume that amylase enzymes bind starch at random positions on the linear chain (e.g., the single-attack model [53]) and cut it into two smaller oligosaccharides, none of which are large enough to bind iodine. This single-step enzymatic hydrolysis reaction can be modeled by Michaelis-Menten kinetics, which gives us the classic equation of enzyme catalyzed substrate elimination:

$$\frac{d\left[S\right]}{dt}=-k_{cat}\frac{\left[E\right]\left[S\right]}{K_{M}+\left[S\right]}.$$ (1)

The general solution of this differential equation is:

$$\left[S\left(t\right)\right]=K_{M}W\left[\frac{\left[S\left(0\right)\right]}{K_{M}}e^{\left(\left[S\left(0\right)\right]-k_{cat}\left[E\right]t\right)/K_{M}}\right],$$ (2)

where *W[*.*]* denotes the Lambert function [79,80]. The second model we propose, which we illustrate in Fig 1B, captures the scenario of a longer starch chain, which requires two successive, random-attack enzymatic hydrolysis reactions to be converted into iodine-negative oligosaccharides.

We make the further simplifying assumption that the length of the starch molecule does not substantially affect the catalytic rate of the amylase enzyme, so that both *K*_*M*_ and *k*_*cat*_ are the same for both short and long starch chains. This is justified in that the total number of sites where an endoamylase may cut is approximately the same in both a single long and two shorter starch chains (both differ by just one binding site, so for a sufficiently long chain the difference between the two is negligible). This assumption would fail to capture certain alternative mechanisms, such as the “multiple attack” model [53]. However, as we shall see, the model ensuing from this assumption provides an excellent fit to the data (Fig 1C and 1D) and has a very strong predictive power (Fig 1E and 1F), suggesting that it captures reasonably well the mechanism of enzymatic starch hydrolysis in our experiments, at least at this coarse-grained level. The model takes the form:

$$\frac{d}{dt}{\left[S\right]}_{long}=-k_{cat}\frac{\left[E\right]{\left[S\right]}_{long}}{K_{M}+{\left[S\right]}_{long}},$$ (3)

$$\frac{d}{dt}{\left[S\right]}_{short}=k_{cat}\frac{\left[E\right]{\left[S\right]}_{long}}{K_{M}+{\left[S\right]}_{long}}-k_{cat}\frac{\left[E\right]{\left[S\right]}_{short}}{K_{M}+{\left[S\right]}_{short}},$$ (4)

where [S]_long_ and [S]_short_ are the concentrations of long and short chains of starch, respectively. The full solution to this model is cumbersome [80]. However, previous characterization of *K*_*M*_ for various *Bacillus*-secreted amylases acting on nonsoluble starch are in the range of approximately 2–15 mg/mL [81–84], whereas the initial starch concentration in our experiments is 1 mg/mL. Therefore, during our incubation reactions, the starch concentration [S] will be generally lower than the expected *K*_*M*_. Taking the limit [*S*] << *K*_*M*_ (where [*S*] = [*S*]_long_ + [*S*]_short_), the system of differential equations above takes the simpler form:

$$\frac{d}{dt}[S{]}_{long}=-\frac{k_{cat}\left[E\right]}{K_{M}}[S{]}_{long},$$ (5)

$$\frac{d}{dt}[S{]}_{short}=\frac{k_{cat}\left[E\right]}{K_{M}}[S{]}_{long}-\frac{k_{cat}\left[E\right]}{K_{M}}{\left[S\right]}_{short}$$ (6)

The concentration of starch as a function of time can be readily found by solving this system of equations using standard techniques of calculus and then adding the two solutions to obtain [*S(t)*] = [*S(t)*]_long_ + [*S(t)*]_short_:

$$\left[S\left(t\right)\right]=\left[S\left(0\right)\right]\left(1+k_{cat}\left[E\right]t/K_{M}\right)e^{-k_{cat}\left[E\right]t/K_{M}}$$ (7)

In our experiments, the two parameters we can externally control are the incubation time (*t*) and the concentration of enzyme ([*E*]) added to the starch solution. We note, as we do in the text, that both of these always appear multiplying one another in all of the models presented above. Therefore, the natural variable that governs the amount of starch degraded is the product of [*E*] and *t*. For convenience, we can define the parameter *x* = ([*E*]/[*E*_0_]), which represents the enzyme dilution (relative to the reference stock concentration [*E*_*0*_]) and the maximum reaction velocity *V* = *k*_*cat*_ *[E*_*0*_*]*/*K*_*M*_. Defining the variable *z* = *x⋅t*, the fraction of starch hydrolyzed, after a time *t*, by a dilution *x* from an enzyme of velocity *V* at stock concentration [*E*_*0*_] can thus be written as:

$$f_{sta}\left(t\right)=1-\frac{K_{M}}{\left[S\left(0\right)\right]}W\left[\frac{\left[S\left(0\right)\right]}{K_{M}}e^{\left(\left[S\left(0\right)\right]-k_{cat}\left[E_{0}\right]xt\right)/K_{M}}]=1-\frac{K_{M}}{\left[S\left(0\right)\right]}W[\frac{\left[S\left(0\right)\right]}{K_{M}}e^{\left(\left[S\left(0\right)\right]-k_{cat}\left[E_{0}\right]z\right)/K_{M}}\right]$$ (8)

for the one-step Michaelis-Menten model and as

$$f_{sta}\left(z\right)=1-\left[S\left(z\right)\right]/\left[S\left(0\right)\right]=1-\left(1+Vz\right)e^{-Vz}$$ (9)

for the two-step model described above, where the single fitting parameter is *V = k*_*cat*_ *[E*_*0*_*]*/*K*_*M*_. The two scenarios (captured by Eqs 8 and 9, respectively) differed in whether one or two sequential cleavage steps are required to break down a starch polymer into smaller oligosaccharides that are too short to cause a colorimetric signal in an iodine test [53]. In both scenarios, we model each enzymatic cleavage reaction by Michaelis-Menten kinetics using parameters *k*_*cat*_ (the catalytic rate per enzyme) and *K*_*M*_ (the Menten constant). In the two-step model (Fig 1B), we also made the approximation that both steps had equal rates (*V* = *k*_*cat*_[*E*_*0*_]/*K*_*M*_) and that the starch concentration [*S*] was much smaller than *K*_*M*_. This approximation reflects our experimental conditions. For both models, we calculated the fraction of starch degraded (*f*_*sta*_) by an enzyme of concentration [*E*_*0*_] diluted by a factor *x* and incubated with a starch concentration [*S*(0)] for a period of time *t*. Although both models exhibit a similar qualitative sigmoidal shape, both predict different quantitative dependencies between *f*_*sta*_ and the product of *x* and *t*, which we denote as *z*. Therefore, both models can be discriminated on a quantitative basis.

To test the performance of these two models, we fitted them to the results of a starch hydrolysis experiment, in which we incubated commercially available, purified *B*. *subtilis* amylase (Sigma-Aldrich, Cat. No. 10070-10G) at various concentrations (from 0.4 to 100 μg/mL in 2X increments) with a 1 mg/mL soluble starch solution (prepared as explained in the previous section) and incubated for various times (0, 10, 20, 30, 60, 120, 240 min) at T = 30°C. Both models were fitted using the NonLinearModelFit function in Mathematica (Wolfram). The two-step model fits the data better (Akaike information criterion [AIC] = −262.5, versus AIC = −238.8 for the one-step model) despite having just one fitting parameter (*V* = *k*_*cat*_[*E*_*0*_]/*K*_*M*_) as opposed to two (*k*_*cat*_, *K*_*M*_), as can be also readily seen by visual inspection of both the fit (Fig 1B) and the residuals (S1 Fig). All other fits to kinetic data were performed using the *nls* function in R.

To extend this model to cocktails of multiple enzymes, we resort to the theory of chemical kinetics, which predicts that when a reaction can occur independently through multiple parallel channels, the overall rate of the reaction is the sum of the rates for each of the independent channels [85,86]. In our model, each of the two reaction steps can be catalyzed by the *m* types of enzymes in the cocktail. Since all enzymes break down starch independently from each other, the velocity of each step when enzymes from *m* different monocultures are mixed is given by:

$$V_{m}=\overset{m}{\underset{j=1}{\sum }}V_{j}{}^{\left(0\right)}=\overset{m}{\underset{j=1}{\sum }}(k_{cat}{}^{j}/K_{M}{}^{j}){\left[E_{0}\right]}_{j},$$ (10)

where *V*_*j*_^*(0)*^ *= (k*_*cat*_^*j*^*/K*_*M*_^*j*^)[*E*_0_]_j_ represents the velocity of the reaction catalyzed by the enzymes released by species *j* after 24 hr of growth in monoculture. The null biochemical model in the text is therefore given by Eq 10. Entering the first equality in Eq 10 into Eq 9, we can calculate the fraction of starch degraded by a cocktail of enzymes *j* = 1, … *n*_*s*_ as:

$$f_{sta}\left(z\right)=1-\left(1+\overset{m}{\underset{j=1}{\sum }}V_{j}{}^{\left(0\right)}z\right)e^{-\overset{m}{\underset{j=1}{\sum }}V_{j}{}^{\left(0\right)}z}.$$ (11)

Eq 11 gives us the fraction of starch degraded over a time *t* by a cocktail of enzymes of velocities *V*_*j*_^*(0)*^ that act independently on the substrate and whose catalytic rates are not affected by each other’s presence. It is important to emphasize that when multiple species are grown in coculture, Eq 11 does not necessarily apply, since ecological interactions can in principle alter the amount of enzymes released by each species, as well as their activity. We also assume throughout that the enzymes from each species are not necessarily identical and that they can have different biochemical velocities.

### Determining pairwise and third-order interactions

HOIs can be quantified by following the scheme in Fig 3A and are briefly summarized below. Let us consider a consortium of three species (A,B,C) and denote it by *V*_*ABC*_^*(0)*^, the function of this consortium in the absence of any other members in the community (which, consistent with the notation we followed in this paper, is denoted by the ^(0)^ superscript). In what follows, the function of interest will be the collective starch hydrolysis rate of the enzymes secreted by the consortium. We can decompose *V*_*ABC*_^*(0)*^ in terms of (1) the functional contributions from each of the single species in isolation *V*_*j*_^*(0)*^ (where *j = A*,*B*,*C*), which are equal to the catalytic rates of the enzymes secreted by each species in monoculture; (2) the pairwise interactions measured from each pair in isolation (Δε_ij_^(0)^, where *i*,*j = A*,*B*,*C*); and (3) a term that captures the HOIs when the three species are together in coculture (Δε_ABC_^(0)^):

$$V_{ABC}{}^{\left(0\right)}=\underset{i}{\sum }V_{i}{}^{\left(0\right)}+\underset{i\neq j}{\sum }\varepsilon_{ij}{}^{\left(0\right)}+\Delta \varepsilon_{ABC}{}^{\left(0\right)}i,j=A,B,C.$$ (12)

*V*_*ABC*_^*(0)*^ is the net function of the three-species community, which can be measured experimentally. In addition, all of the *V*_*i*_^*(0)*^ and ε_ij_^(0)^ parameters can also be obtained independently from the function of the monocultures and pairwise cocultures. Therefore, we can experimentally determine the strength of three-way interactions (Δε_ABC_^(0)^) as a function of independently measured experimental parameters:

$$\Delta \varepsilon_{ABC}{}^{\left(0\right)}=V_{ABC}{}^{\left(0\right)}-\underset{i}{\sum }V_{i}{}^{\left(0\right)}-\underset{i\neq j}{\sum }\varepsilon_{ij}{}^{\left(0\right)}i,j=A,B,C.$$ (13)

Propagation of errors was used to estimate the uncertainty in the measurement of pairwise and third-order interactions through this approach.

### Determining fourth- and higher-order interactions

For communities of size *M* (larger than three species), Eq 12 can be generalized as

$$V_{\vec{M}}{}^{\left(0\right)}=\overset{M}{\underset{i=1}{\sum }}V_{i}{}^{\left(0\right)}+\overset{M}{\underset{i\neq j}{\sum }}\varepsilon_{ij}{}^{\left(0\right)}+H_{3}{}^{\left(0\right)}+H_{4}{}^{\left(0\right)}+H_{5}{}^{\left(0\right)}+\cdots ,$$ (14)

where the collection of HOIs can be defined as the sum of all of the three-way (*H*_3_^(0)^), four-way (*H*_4_^(0)^), five-way (*H*_5_^(0)^) interactions in isolation, etc., and can be determined experimentally as:

$$H_{\vec{M}}{}^{\left(0\right)}=\overset{M}{\underset{S=3}{\sum }}H_{S}{}^{\left(0\right)}=V_{\vec{M}}{}^{\left(0\right)}-\overset{M}{\underset{i=1}{\sum }}V_{i}{}^{\left(0\right)}-\overset{M}{\underset{i\neq j}{\sum }}\varepsilon_{ij}{}^{\left(0\right)}.$$ (15)

We can construct approximations to ${V_{\vec{M}}}^{\left(0\right)}$ that take into account only pairwise interactions, or only up to third-order interactions, as:

$$\Phi_{\vec{M}}{}^{\left(2\right)}=\overset{M}{\underset{i=1}{\sum }}V_{i}{}^{\left(0\right)}+\overset{M}{\underset{i\neq j}{\sum }}\varepsilon_{ij}{}^{\left(0\right)},$$ (16)

or

$$\Phi_{\vec{M}}{}^{\left(3\right)}=\overset{M}{\underset{i=1}{\sum }}V_{i}{}^{\left(0\right)}+\overset{M}{\underset{i\neq j}{\sum }}\varepsilon_{ij}{}^{\left(0\right)}+H_{3}{}^{\left(0\right)}.$$ (17)

### Minimal model of constitutive amylase expression

To formulate a minimal model of amylase production that incorporates population growth, we first made the simplifying assumption that the extracellular amylase is constitutively expressed by each cell at a constant rate μ_exp_. This is a standard and widely used null model of gene expression [87–90]. Assuming that the population grows exponentially with growth rate *r*, our model can be written through the coupled differential equations:

$$\frac{dN}{dt}=rN\left(t\right),$$ (18)

$$\frac{da}{dt}=\mu_{exp}N\left(t\right)$$ (19)

where *a*(*t*) represents the amount of amylase in the environment. From Eq 18, we find that *N*(*t*) = *N*(0) e^*r t*^. Entering this into Eq 19 and integrating it over the incubation time T, we find that the total amount of enzyme released into the environment (*E*) is given by *E* = (μ_exp_/*r*)(*N*(*T*) − *N*(0)) = (μ_exp_/*r*)*ΔN*.

The proportionality between *E* and the total population growth *ΔN* does not require exponential growth and is also found in a model in which the population switches to stationary phase at time τ < *T*. To prove this, let us assume that cells express amylase constitutively at rate μ_exp_ in exponential phase and (also constitutively) at rate μ_sta_ at stationary phase. The total amount of amylase produced is the sum of the amylase produced in each phase of growth: *E* = *E*_exp_ + *E*_sta_. Following the argument given above, *E*_exp_ = (μ_exp_/r)(*N*(τ) − *N*(0)). In stationary phase (for t > τ), *N*(t) = *N*(τ) and the differential equation that describes amylase production reduces to d*a*/dt = μ_sta_*N*(τ). Integrating this equation between *a* = [0,*E*] and *t* = [τ,*T*], we find *E*_sta_ = μ_sta_*N*(τ)(*T −* τ). Since the population size is constant in stationary phase, *N*(τ) = *N*(*T*) and we can write *E*_sta_ as *E*_sta_ = μ_sta_*N*(*T*)(*T −* τ). Given that *N*(*T*) >> *N*(0) (a limit that is fulfilled in our experiments), we can write *N*(*T*) ≈ *ΔN*, and we approximate *E*_sta_ = μ_sta_(*T −* τ)*N*(*T*) ≈ μ_sta_(T − τ)*ΔN*. Adding up *E*_sta_ and *E*_exp_, we find:

$$E=\left(\mu_{exp}/r\right)\Delta N+\mu_{sta}\left(T-\tau \right)\Delta N=\left[\left(\mu_{exp}/r\right)+\mu_{sta}\left(T-\tau \right)\right]\Delta N.$$ (20)

We note that μ_exp_/*r* is the ratio of the rate of amylase expression and the rate of growth. This ratio is equal to the average amount of amylase produced by a single cell over a doubling period *t*_div_ = 1/*r*. In turn, μ_sta_(*T −* τ) represents the total amount of amylase produced per cell during stationary phase. Given that *N*(*T*) >> *N*(0), we conclude that under constitutive expression, the cumulative amount of amylase secreted by each species is proportional to that species’ population size at the time of harvest, which as discussed in the main text we denote as *N*_i_(*T*) ≡ *n*_i_. Under the Michaelis-Menten model above, the amylolytic rate is proportional to the enzyme concentration. Therefore, we can write the function of a species as *V*_i_ = *λ*_i_*n*_i_. Under our additive null model, the function of an *M*-species consortium would thus be written as:

$$V_{\vec{M}}{}^{\left(0\right)}=\overset{M}{\underset{i=1}{\sum }}\lambda_{i}n_{i}.$$ (21)

### Minimal model of amylase expression under negative-feedback regulation

We capture this negative-feedback regulation through a simple phenomenological model (Fig 5D and 5E), which coarse-grains the complex biochemical interactions and gene-regulatory circuitry that controls amylase expression through a phenomenological Hill function in which the expression rate of extracellular amylase is down-regulated by the amount of amylase already accumulated in the environment. Such approach is routine practice in the modeling of gene-regulatory circuits [58,88,89], including those mediated by catabolite repression [57,90–92]. To make this model as unconstrained as possible, we analyze a general form of the phenomenological Hill function (equivalent to the Holling type III functional response) that captures a large range of scenarios including different degrees of cooperativity and amplitude of the negative-feedback function. Assuming first that the population is growing exponentially, our model takes the form:

$$\frac{dN}{dt}=rN\left(t\right),$$ (22)

$$\frac{da}{dt}=\mu_{exp}N\left(t\right)\frac{1}{1+{\left(a/A\right)}^{q}}$$ (23)

Here, *q* characterizes the steepness of the negative feedback (e.g., if *q* >> 1, it will be switch-like, whereas if *q* = 1, it will be gradual), and *A* represents the threshold concentration of extracellular amylase above which the amylase gene is turned off. The relationship between *ΔN* and *E* can be obtained by integrating both equations, and by defining the threshold population size N* = *A*/(*t*_div_μ_exp_), we obtain:

$$\Delta N=[E/A+\frac{1}{q+1}\left(E/A{)}^{q+1}\right]N^{*}.$$ (24)

We define *H* as the inverse function of *f*[x] = x + x^*q*+1^/(*q* + 1), which gives us:

$$E/A=H\left[\Delta N/N^{*}\right].$$ (25)

As shown in S6 Fig for various values of *q*, *E* is a saturating function of *ΔN*. In other words, the model predicts that the amylolytic rate of a population becomes insensitive to changes in the size of the population, past certain population threshold. Also shown in S6 Fig, for comparison, is an example of the type II saturating function we use throughout the paper.

Although we have derived this result for an exponentially growing population, it is straightforward to generalize this result to the same case studied in the previous section: a population that switches to stationary phase at time *τ* < *T* after growing exponentially between 0 and *τ*. Integrating Eq 23, we find:

$$\int_{0}^{T}N\left(t\right)dt=\left(A/\mu \right)[E/A+\frac{1}{q+1}\left(E/A{)}^{q+1}\right].$$ (26)

Defining *t*_*div*_ as the timescale of cell division (where *r* = 1/*t*_*div*_), the first half of Eq 26 can be written as:

$$\int_{0}^{\tau }N\left(0\right)e^{t/t_{div}}dt+\overset{T}{\underset{\tau }{\int }}N\left(\tau \right)dt\approx \Delta N\left(t_{div}+T-\tau \right).$$ (27)

This gives us a relationship between *E* and *ΔN*:

$$\Delta N=[E/A+\frac{1}{q+1}\left(E/A{)}^{q+1}\right]N^{\#},$$ (28)

which has the same form as Eq 24, only now the threshold population size is given by *N*^#^≈*A*/[μ(*T–* τ + *t*_*div*_)]. As we show in S6 Fig, this model predicts that the total amount of amylase produced by a population saturates when the population size grows beyond a threshold population size.

### Separating the contribution of behavioral and population dynamics pairwise interactions

In Fig 3, we define the functional interactions between species A and B as *ε*_AB_^(0)^, where:

$$V_{AB}{}^{\left(0\right)}=V_{A}^{\left(0\right)}+V_{B}^{\left(0\right)}+\varepsilon_{AB}^{\left(0\right)}.$$ (29)

Here, *V*_*AB*_^(0)^ is the measured function of the consortium formed by species A and species B, and *V*_*A*_^(0)^ and *V*_*B*_^(0)^ represent the measured function of each of the species in their respective monocultures. In Fig 5, we introduced and quantified a second null model that incorporated the effect of population dynamics. This new model made use of the functions *V*_*A*_(.) and *V*_*B*_(.), which quantitatively map the population size of species A and B after 24 hr of growth in monoculture with the amylolytic rate of the enzymes released by each population over that period. These functions were estimated for all species as shown in Figs 5C and S7. Let us denote the population size reached by species A and B in monoculture as *n*_A_^(0)^ and *n*_B_^(0)^ and the population size of A and B in coculture as *n*_*A*_^*(AB)*^ and *n*_*B*_^*(AB)*^. Employing this notation, Eq 29 can be rewritten as:

$$V_{AB}{}^{\left(0\right)}=V_{A}\left(n_{A}^{\left(0\right)}\right)+V_{B}\left(n_{B}^{\left(0\right)}\right)+\varepsilon_{AB}^{\left(0\right)}.$$ (30)

According to the second null model that incorporates population dynamics, the function of this consortium can also be related to the population size of A and B after 24 hr of coculture, as:

$$V_{AB}{}^{\left(0\right)}=V_{A}\left(n_{A}^{\left(AB\right)}\right)+V_{B}\left(n_{B}^{\left(AB\right)}\right)+\varepsilon_{AB}^{behav},$$ (31)

where $\varepsilon_{AB}^{behav}$ represents the deviation from the null model and can be interpreted as capturing the effect of changes in the expression of amylase. This is the interaction that is quantified in Fig 6. It is straightforward to rewrite Eq 31 by adding and subtracting *V*_*A*_(*n*_A_^0^) and *V*_*B*_(*n*_B_^0^) to the right side:

$$V_{AB}{}^{\left(0\right)}=\left(V_{A}\left(n_{A}^{\left(AB\right)}\right)-V_{A}\left(n_{A}^{\left(0\right)}\right)\right)+\left(V_{B}\left(n_{B}^{\left(AB\right)}\right)-V_{B}\left(n_{B}^{\left(0\right)}\right)\right)+V_{A}\left(n_{A}^{\left(0\right)}\right)+V_{B}\left(n_{B}^{\left(0\right)}\right)+\varepsilon_{AB}^{behav}.$$ (32)

We can then define ${\delta_{A}}^{\left(AB\right)}=V_{A}\left(n_{A}^{\left(AB\right)}\right)-V_{A}\left(n_{A}^{\left(0\right)}\right)$ as the change in amylolytic function of A that would be caused solely by an increase or decrease in its population size in coculture with B. We can similarly define ${\delta_{B}}^{\left(AB\right)}=V_{B}\left(n_{B}^{\left(AB\right)}\right)-V_{B}\left(n_{B}^{\left(0\right)}\right)$ and the sum of both as:

$$\varepsilon_{AB}^{pop}=\left(V_{A}\left(n_{A}^{\left(AB\right)}\right)-V_{A}\left(n_{A}^{\left(0\right)}\right)\right)+\left(V_{B}\left(n_{B}^{\left(AB\right)}\right)-V_{B}\left(n_{B}^{\left(0\right)}\right)\right)=\delta_{A}{}^{\left(AB\right)}+\delta_{B}{}^{\left(AB\right)}.$$ (33)

Entering Eq 33 into Eq 32, we get:

$$V_{AB}{}^{\left(0\right)}=V_{A}\left(n_{A}^{\left(0\right)}\right)+V_{B}\left(n_{B}^{\left(0\right)}\right)+\varepsilon_{AB}^{pop}+\varepsilon_{AB}^{behav}.$$ (34)

It is therefore straightforward to see, by comparing Eq 34 with Eq 30, that the total interaction we reported in Fig 2 from comparison to the P/A null model ($\varepsilon_{AB}^{\left(0\right)}$) is the sum of an interaction that is solely due to changes in population dynamics in coculture relative to monoculture ($\varepsilon_{AB}^{pop}$) (S11 Fig) and an interaction ($\varepsilon_{AB}^{behav}$) that captures the overall of change in amylase expression for both species in coculture relative to monoculture (S11 Fig):

$$\varepsilon_{AB}^{\left(0\right)}=\varepsilon_{AB}^{behav}+\varepsilon_{AB}^{pop}.$$ (35)

The result in Eq 35 showcases the connection between the interactions as defined from both null models, and it allows us to estimate the contribution of population dynamics to the net interactions reported in Fig 2. It is worth noting that whereas $\varepsilon_{AB}^{pop}$ represents an interaction that is independent of any changes in behavior or gene expression, $\varepsilon_{AB}^{behav}$ is defined at a given final population size for both *A* and *B*, so it is not entirely independent of changes in population dynamics.

### Determining high-order behavioral interactions using the revised null model

Recalling our definition of ${H_{\vec{M}}}^{\left(0\right)}$ as the sum of all HOIs for an *M-*species consortium, we can rewrite the function of an *M-*species consortium (which we denote by ${V_{\vec{M}}}^{\left(0\right)})$ as:

$$V_{\vec{M}}{}^{\left(0\right)}=\overset{M}{\underset{i=1}{\sum }}V_{i}{}^{\left(0\right)}+\overset{M}{\underset{i\neq j}{\sum }}\varepsilon_{ij}{}^{\left(0\right)}+H_{\vec{M}}{}^{\left(0\right)}.$$ (36)

It is straightforward to show per our definition above that ${{V_{i}}^{\left(0\right)}=V}_{i}\left(n_{i}^{\left(0\right)}\right)=\left(V_{i}\left({n_{i}}^{\left(\vec{M}\right)}\right)-\delta_{i}^{\left(\vec{M}\right)}\right)$, where ${n_{i}}^{\left(\vec{M}\right)}$ is the density reached by species *i* in coculture with the *M-*species consortium, and $\delta_{i}^{\left(M\right)}$ is the change in function of species *i* one would predict just due to the different population growth of species *i* when embedded in the *M*-species consortium relative to its monoculture. From Eq 35, we also know that $\varepsilon_{ij}^{\left(0\right)}=\varepsilon_{ij}^{behav}+\varepsilon_{ij}^{pop}$. This all allows us to rewrite Eq 36 as:

$$V_{\vec{M}}{}^{\left(0\right)}=\sum_{i=1}^{N}V_{i}\left(n_{i}{}^{\left(\vec{M}\right)}\right)+\sum_{i\neq j}^{M}\varepsilon_{ij}{}^{behav}+\sum_{i\neq j}^{M}\varepsilon_{ij}{}^{pop}-\sum_{i=1}^{M}\delta_{i}^{\left(\vec{M}\right)}+H_{\vec{M}}{}^{\left(0\right)}.$$ (37)

We can then redefine the combined high-order behavioral interactions (${H_{\vec{M}}}^{behav}$) as the deviation from the revised null model that incorporates population dynamics after adding to it also the sum of all pairwise behavioral interactions, i.e.:

$$V_{\vec{M}}{}^{\left(0\right)}=\sum_{i=1}^{M}V_{i}\left(n_{i}{}^{\left(\vec{M}\right)}\right)+\sum_{i\neq j}^{M}\varepsilon_{ij}{}^{behav}+H_{\vec{M}}{}^{behav},$$ (38)

where

$$H_{\vec{M}}{}^{behav}=H_{\vec{M}}{}^{\left(0\right)}+\sum_{i\neq j}^{M}\varepsilon_{ij}{}^{pop}-\sum_{i=1}^{M}\delta_{i}^{\left(\vec{M}\right)}.$$ (39)

This allows us to separate the effect of population dynamics and behavioral contributions to the total HOIs:

$$H_{\vec{M}}{}^{\left(0\right)}=H_{\vec{M}}{}^{behav}+H_{\vec{M}}{}^{pop},$$ (40)

where we have defined the contribution of population dynamics to higher-order interactions as:

$$H_{\vec{M}}{}^{pop}=\left(\sum_{i=1}^{M}\delta_{i}^{\left(\vec{M}\right)}-\sum_{i\neq j}^{M}\varepsilon_{ij}{}^{pop}\right).$$ (41)

It may be difficult to appreciate this last point using such a compact, general notation. To illustrate it more clearly, consider the particular case of three-species consortium containing species A, B, and C, in which there are no behavioral interactions. In this instance, ${H_{\vec{M}}}^{pop}={H_{ABC}}^{pop}$. Expanding Eq 41 for this consortium, we would get:

$$H_{ABC}{}^{pop}=\delta_{A}^{\left(ABC\right)}+\delta_{B}^{\left(ABC\right)}+\delta_{C}^{\left(ABC\right)}-\varepsilon_{AB}{}^{pop}-\varepsilon_{BC}{}^{pop}-\varepsilon_{AC}{}^{pop}.$$ (42)

We can further expand the pairwise population dynamics interactions as the sum of the effect on each species as discussed above:

$$H_{ABC}{}^{pop}=\delta_{A}^{\left(ABC\right)}+\delta_{B}^{\left(ABC\right)}+\delta_{C}^{\left(ABC\right)}-\left(\delta_{A}^{\left(AB\right)}+\delta_{B}^{\left(AB\right)}\right)-\left(\delta_{B}^{\left(BC\right)}+\delta_{C}^{\left(BC\right)}\right)-\left(\delta_{A}^{\left(AC\right)}+\delta_{C}^{\left(AC\right)}\right),$$ (43)

which can be rewritten as:

$$H_{ABC}{}^{pop}=\left(\delta_{A}^{\left(ABC\right)}-\left(\delta_{A}^{\left(AB\right)}+\delta_{A}^{\left(AC\right)}\right)\right)+\left(\delta_{B}^{\left(ABC\right)}-\left(\delta_{B}^{\left(AB\right)}+\delta_{B}^{\left(BC\right)}\right)\right)+\left(\delta_{C}^{\left(ABC\right)}-\left(\delta_{C}^{\left(BC\right)}+\delta_{C}^{\left(AC\right)}\right)\right).$$ (44)

Consistent with our notation above, $\delta_{A}^{\left(ABC\right)}$ reflects the increase (decrease) in the amount of amylase produced by species A due to larger (lower) population growth when in coculture with B and C. In turn, $\delta_{A}^{\left(AB\right)}$ and $\delta_{A}^{\left(AC\right)}$ would reflect the change in amylase production by species A due to changes to its population dynamics when in coculture with either B alone or C alone, respectively. Each term within parentheses characterizes the deviation between the observed change in amylase production by a member of the three-species consortium and the prediction from summing up the deviations observed in each combinatorially assembled two-species subconsortium.

In Fig 6A, we estimated $\varepsilon_{AB}^{pop}$ for all pairs using Eq 33. Error bars were determined by error propagation. In Fig 6B, we estimated ${H_{\vec{M}}}^{pop}$ from Eq 41, and error bars were also determined by error propagation of the terms of the equation.

### Estimating model parameters

For *P. polymyxa*, we estimated the saturating population size *K*_*i*_ by fitting the data in Figs 5C and S7 to the model using R function *nls*. The monocultures supplemented with *B*. *megaterium* supernatant were discarded because of inconsistencies among the replicates. For the other *Bacillus* species, we estimated *u*_*i*_ by fitting the data in S7 Fig to a constant value. The value of *B*. *mojavensis* is likely overestimated, as it reaches higher function values when grown supplemented with spent media of the other species, increasing monotonically with the amount added (S7 and S8 Figs). The value of *K*_*i*_ could not be estimated from this data directly, since the population size is in all cases much larger than *K*_*i*_ (e.g., the data are flat and does not show curvature). The maximum bound of this parameter could be conservatively estimated as half of the minimum population size in our spent media addition experiments.

### Quantifying population size of the different *Bacillus* species in consortia

Combinatorially assembled communities were incubated at 30°C for 24 hr and then stored at −80°C in 40% glycerol. To measure the amount of *P. polymyxa* CFUs in each mixture, approximately 20 μl of the frozen stock was melted and serially diluted 1:10 up to 1:10^5^. Fifty microliters of dilutions 1:10^2^, 1:10^3^, 1:10^4^, and 1:10^5^ were then plated onto BE Starch and incubated at 30°C for 48 hr. Plates were scanned with an EPSON Perfection V700-V750 scanner at a 300-dpi resolution, and CFU/mL was recorded with the ImageJ plugin Cell Counter. Colony morphology was different enough to easily allow differential identification of all species (S14 Fig).

### Cross-feeding assays

The six *Bacillus* strains were individually grown in 3 mL of 1x bSAM at 30°C for 24 hr. To obtain the supernatants for cross-feeding assays, cultures were pelleted in 15-mL conical tubes at 3,500 rpm for 15 min in an Eppendorf 5810 tabletop centrifuge. The supernatants were carefully transferred to clean, sterile tubes and kept at room temperature. To eliminate any cells, supernatants were transferred to 0.2-μm spin-filter columns (VWR 82031–358) and centrifuged at 14,000*g* for 5 min. Each flow-through was subjected to a second filtration step through a 30-KDa centrifugal device (Nanosep 3K; Pall OD003C33) at 5,000*g* for 10 min to avoid any amylases in subsequent steps. All spent media were sequentially diluted 1:2 in water, and 50 μL was mixed with 250 μL 2x bSAM and 200 μL water in 96-deep-well plates to obtain 1x SAM supplemented at a final concentration of 0.1, 0.05, 0.025, 0.0125, 0.00625, and 0. Two microliters containing 10^6^ CFU of each of the six *Bacillus* species, processed as described above, was inoculated into this media and grown still at 30°C for 24 hr in 96-deep-well plates (VWR). Plates were covered with Aerogel film (VWR). Optical density (620 nm) of the grown cultures was measured, and the amylolytic rate was determined as above at the end of the incubation period. Prior to filtering, all devices were sanitized with 70% ethanol, according to manufacturer instructions.

## Supporting information

S1 Fig Comparisons of residuals from one-step and two-step biochemical models. (A) Residuals of both one-step and two-step models fit to the data in Fig 1C. The residuals of the fits in Fig 1C are shown as a function of *z*. Gray line: one step; black line: two-step model. (B) Comparison of two-step model fits for two biological replicates. In the *x* axis, data used for Fig 1; in the *y* axis, data used in Figs 2–5, in the main text.
(TIFF)

S2 Fig Pairwise interactions only marginally correlate to additive expectation. Sum of pairwise interactions (mean ± SE, hr^−1^) is very weakly correlated to additive expectation (mean ± SE, same units) in three-species communities (*ρ* = −0.29, *P* = 0.04). Trios including *P. polymyxa* are shown in orange, and those not including *P. polymyxa* are shown in blue.
(TIFF)

S3 Fig Types of interactions that could affect the function of a microbial consortium. Left panel: Biochemical interactions. The enzymes secreted by different organisms interact with each other producing a higher or lower activity than each would in isolation (e.g., through allosteric interactions or by catalyzing different steps on a complex biochemical pathway). Center panel: Behavioral interactions. Organisms could respond to the presence of other species in their environment by regulating the expression of extracellular enzymes (either inducing or repressing the amount of enzyme produced per cell). Right panel: Population dynamics interactions. Organisms could affect each other’s population size, thereby indirectly affecting the total amount of extracellular enzyme released to the environment.
(TIFF)

S4 Fig The supernatant of different strains filtered through 30-KDa filters shows no amylolytic activity. Supernatant from 24-hr cultures in 1x bSAM supplemented with starch were filtered through either 0.2-μM or 30-KDa filters and assayed for amylolytic activity using Lugol staining (Methods) at 0, 2, and 18 hr. Error bars represent ±SD from three replicates.
(TIFF)

S5 Fig Changes in CFU/mL observed after coculturing bacilli species in pairs. Cultures of the six bacilli species grown at 30°C for 24 hr were concentrated, washed, resuspended in growth media to inoculate monocultures or pairs, and grown at 30°C for an additional 24 hr. Species (light and dark green) are designated as C, *B*. *cereus*; E, *B*. *megaterium*; M, *B*. *mojavensis*; P, *P. polymyxa*; S, *B*. *subtilis*; and T, *B*. *thuringiensis*. Upward arrows denote an increase in CFU/mL from the value obtained when the two species are grown in monocultures to the value obtained after the same two species were cocultured for 24 hr. The experiment was done in two biological replicates of which we measured starch concentration in both and counted CFUs in one. Downward arrows indicate a decrease in CFU/mL. CFU, colony forming unit.
(TIFF)

S6 FigOur minimal model of amylase expression under negative-feedback regulation produces a saturating dependence of the amount of enzyme released and the population size.We plot Eq 24 ($\Delta N/N^{*}=[E/A+\frac{1}{q+1}({E/A)}^{q+1}]$) for various values of *q*: *q* = 4 (light gray), *q* = 8 (dark gray), *q* = 20 (black). By way of comparison, we plot the inverse of a type II Holling function like the one we use in the paper *E*/*A* = (*E*/*A*)_max_ *ΔN*/(N* + *ΔN*) (dashed blue) for (*E*/*A*)_max_ = 2 (the result is not contingent on this choice, and other values gave similar behavior but are not shown for clarity). Inset: log-log scale representation of the same figure.(TIF)

S7 Fig The relation between final population size and function in *Bacilli* species is consistent with a saturating function. We cultured each of the *Bacillus* species in 1x bSAM media supplemented with different amounts of filtered spent media from all of the other taxa (the fraction of the final culture volume made up by the spent media ranged between 0 and 0.1, as shown). After 24 hr of culture, the final population size (in CFU/mL) and the amylolytic function *V* were determined and plotted. With the exception of *P. polymyxa* (Fig 5C, main text) and *B*. *subtilis*, for the whole range of observed population sizes in other *Bacilli* species the function remained approximately flat, consistently with an underlying saturating function. Thus, for these species, we used the average of the observed *V*_*i*_*(n*_*i*_*)* values to estimate parameter *u*_*i*_ (dashed black line; gray indicates ±SD), and we conservatively estimated *K* as one-half of the minimum observed *n*_*i*_ in the data shown in these figures. Using *V* and *K* parameters estimated in this way, we show the resulting saturating function for each species (dashed red lines). CFU, colony forming unit.
(TIFF)

S8 Fig Comparison between the V_i_^(0)^ values obtained by fitting the two-step Michaelis-Menten model to the monocultures and *u*_*i*_ values, i.e., the asymptotic rate using the saturating model. The dashed red line indicates perfect matching. The value of *B*. *mojavensis* is likely overestimated, as it reaches higher function values when grown supplemented with spent media of the other species. The value of *P. polymyxa* is expected not to match, given that V_i_^(0)^ is determined in monoculture and in the absence of any facilitation.
(TIFF)

S9 Fig Population sizes in consortia are above the threshold estimated for the saturating model. The threshold for each species (red line) was conservatively estimated as one-half of the minimal population size in the supernatant conditioning experiment (Figs 5C and S7). The black points represent the CFU/mL counts determined for each species in all of the consortia that it is present. CFU, colony forming unit.
(TIFF)

S10 Fig The predictions from the original presence/absence null model and the predictions from the saturating null model are highly correlated for consortia lacking *P. polymyxa* (ρ = 0.88, *P* < 10^−7^). Each point represents one consortium; error bars represent ±SE.
(TIFF)

S11 FigA combination of metabolic facilitation and amylase expression inhibition explains interactions in *P. polymyxa*.We depict the effect of species B on the amount of enzyme released by species A at different population sizes: (A) An example of how population dynamics interactions may affect the amount of amylase produced by a species: when species B competes with species A, it lowers its population size, but it does not alter the amount of amylase it expresses relative to monoculture. (B) An example of how behavioral interactions may affect the amount of amylase produced by a species: Species B does not affect the growth of species A, but the latter responds to the presence of species B by lowering its investment in amylase production. (C) We propose that the function of communities containing *P. polymyxa* could be explained by a combination of growth facilitation, which pushes the population size over the threshold K_P_, and a behavioral interaction in which *P. polymyxa* reduces its investment in amylase production in coculture with other species. (D) We estimate the lower and upper bound inhibition of the contribution of *P. polymyxa* to community function in mixed culture with other species (i.e., $\delta_{P}^{\vec{M}}$). It is straightforward to show that $\delta_{P}^{\vec{M}}\in [-u_{P}\left(\frac{n_{P}}{n_{P}+K_{P}}\right),{V_{\vec{M}}}^{\left(0\right)}{-u}_{P}\left(\frac{n_{P}}{n_{P}+K_{P}}\right)]$ where, as defined in the text, *u*_*P*_ is the activity of *P. polymyxa* at saturating population size (*n*_*P*_>>*K*_*P*_), and ${V_{\vec{M}}}^{\left(0\right)}$ is the activity of the consortia. The lower bound corresponds to *P. polymyxa* completely suppressing its contribution, and the higher bound corresponds to all other species suppressing their contribution while *P. polymyxa* is the only one secreting amylase.(TIFF)

S12 Fig *P. polymyxa* grows better in media supplemented with vitamins. Optical density at 620 nm of *P. polymyxa* cultures grown at 30°C for 24 hr in rich media (beef extract + 2% w/v starch) or 1× bSAM supplemented or not, as indicated, with 1% vitamin supplement (v/v, ATCC MD-VS). Note that the non-vitamin-supplemented condition (“- Vitamins”) would correspond to the same growth medium (1× bSAM) used in all of the experiments in this paper. This medium (1× bSAM) does not contain vitamins. As expected from prior work [66], addition of a biotin-containing vitamin supplement to bSAM (“+Vitamins”) is sufficient for *P. polymyxa* to grow, as expected. Plots show the media for two replicates and the standard error.
(TIFF)

S13 FigThe strength of high-order interactions increases with community size.(A) Plot showing the prediction of the function (*V*) from a model that includes only up to pairwise interactions against the experimentally measured value for consortia of different richness (represented by point color). (B) Strength of higher-order interactions, measured as the absolute value of the difference between the function determined experimentally (amylolytic activity $V_{\vec{M}}^{\left(0\right)}$, hr^−1^) and the expected one using the model including single and pairwise effects. (C) The strength of high-order interactions increases with community size also when accounting for third-order interactions. Similarly to (B), interactions of order higher than three are measured here as the absolute value of the difference between the function determined experimentally (*V*_*i*_^*(0)*^, hr^−1^) and the expected one using the model including only single, pairwise, and third-order effects. Orange and blue dots represent communities with or without *P. polymyxa*, respectively. All error bars represent ±SE.(TIFF)

S14 Fig Identification of *Bacillus* species based on colony morphology. Combinatorially assembled communities were incubated in 1x bSAM at 30°C for 24 hr and stored at −80°C in 40% glycerol. Approximately 20 μl of the frozen stock was melted and serially diluted 1:10 up to 1:10^5^. Fifty microliters of dilutions 1:10^4^ was plated onto BE Starch and incubated at 30°C for 48 hr. To record colony growth and development, plates were scanned every 24 hr with an EPSON Perfection V700-V750 scanner at a 300-dpi resolution. Colonies were discriminated by naked eye using a Laxco MZS1 Series Stereo Zoom Microscope or the scanned images according to their color, shape, size, roughness of borders, and size of the halo produced upon starch degradation. (A) Agar plate showing the community composed of *B*. *cereus*, *B*. *mojavensis*, *P. polymyxa*, *B*. *subtilis*, and *B*. *thuringiensis*. Insets A1–A4 have been scaled up to show the different colony morphologies found. Arrows point to each of the five *Bacillus* species in this consortium. (B) Agar plate showing the community composed of *B*. *cereus*, *B*. *megaterium*, *P. polymyxa*, *B*. *subtilis*, and *B*. *thuringiensis*. Insets B1–B4 have been scaled up to show the different colony morphologies found. Arrows point to each of the five *Bacillus* species in this consortium. Species are designated as C, *B*. *cereus*; E, *B*. *megaterium*; M, *B*. *mojavensis*; P, *P. polymyxa*; S, *B*. *subtilis*; and T, *B*. *thuringiensis*.
(TIF)

## Abbreviations

CFU: colony forming unit
HOI: high-order interaction
MW: molecular weight
P/A: presence/absence
RMSD: root-mean-square deviation
