# Supplementary figures and images for: High-order interactions distort the functional landscape of microbial consortia
Source: PLoS Biol. 2019 Dec 12;17(12):e3000550. doi: 10.1371/journal.pbio.3000550 (PMC6932822; doi:10.1371/journal.pbio.3000550)

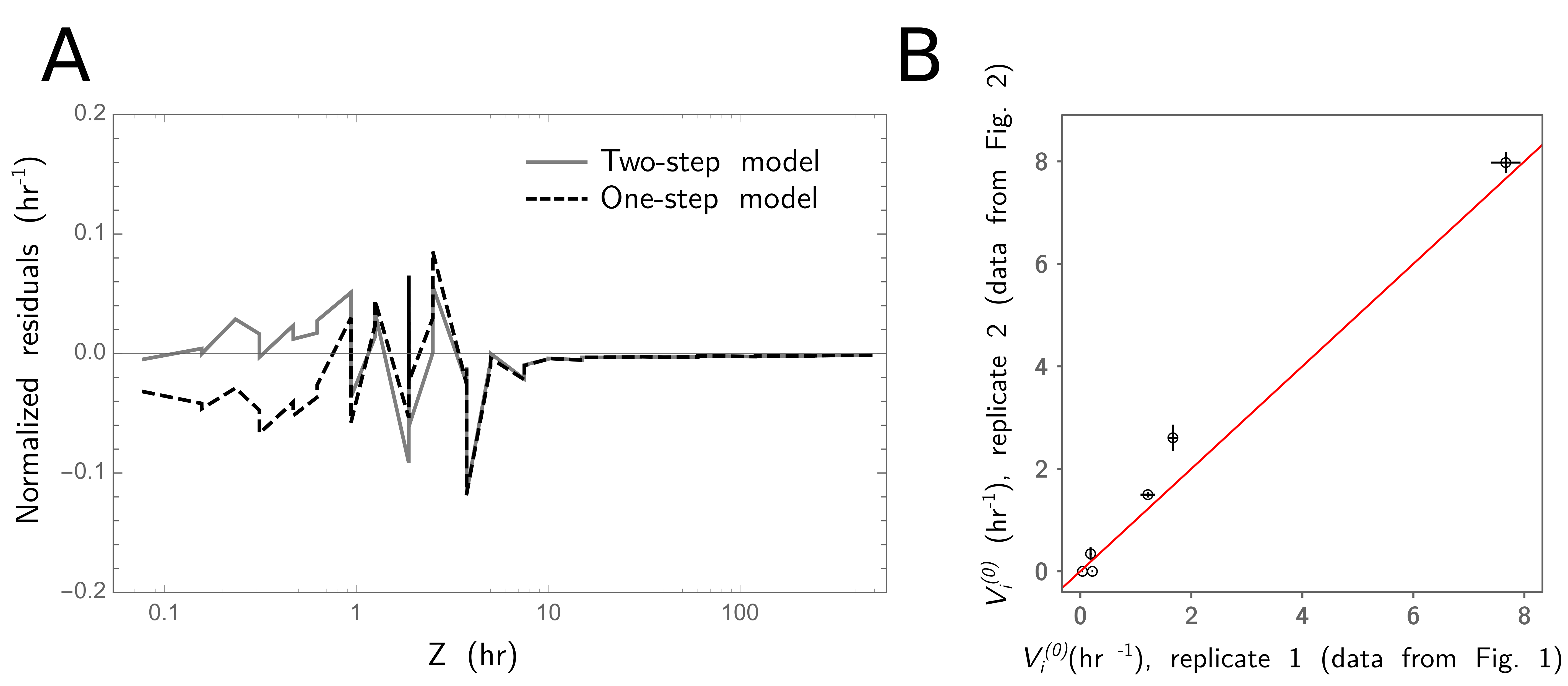

Supplement: S1 Fig — (A) Residuals of both one-step and two-step models fit to the data in Fig 1C. The residuals of the fits in Fig 1C are shown as a function of z. Gray line: one step; black line: two-step model. (B) Comparison of two-step model fits for two biological replicates. In the x axis, data used for Fig 1; in the y axis, data used in Figs 2–5, in the main text. (TIFF) [file pbio.3000550.s001.tiff]

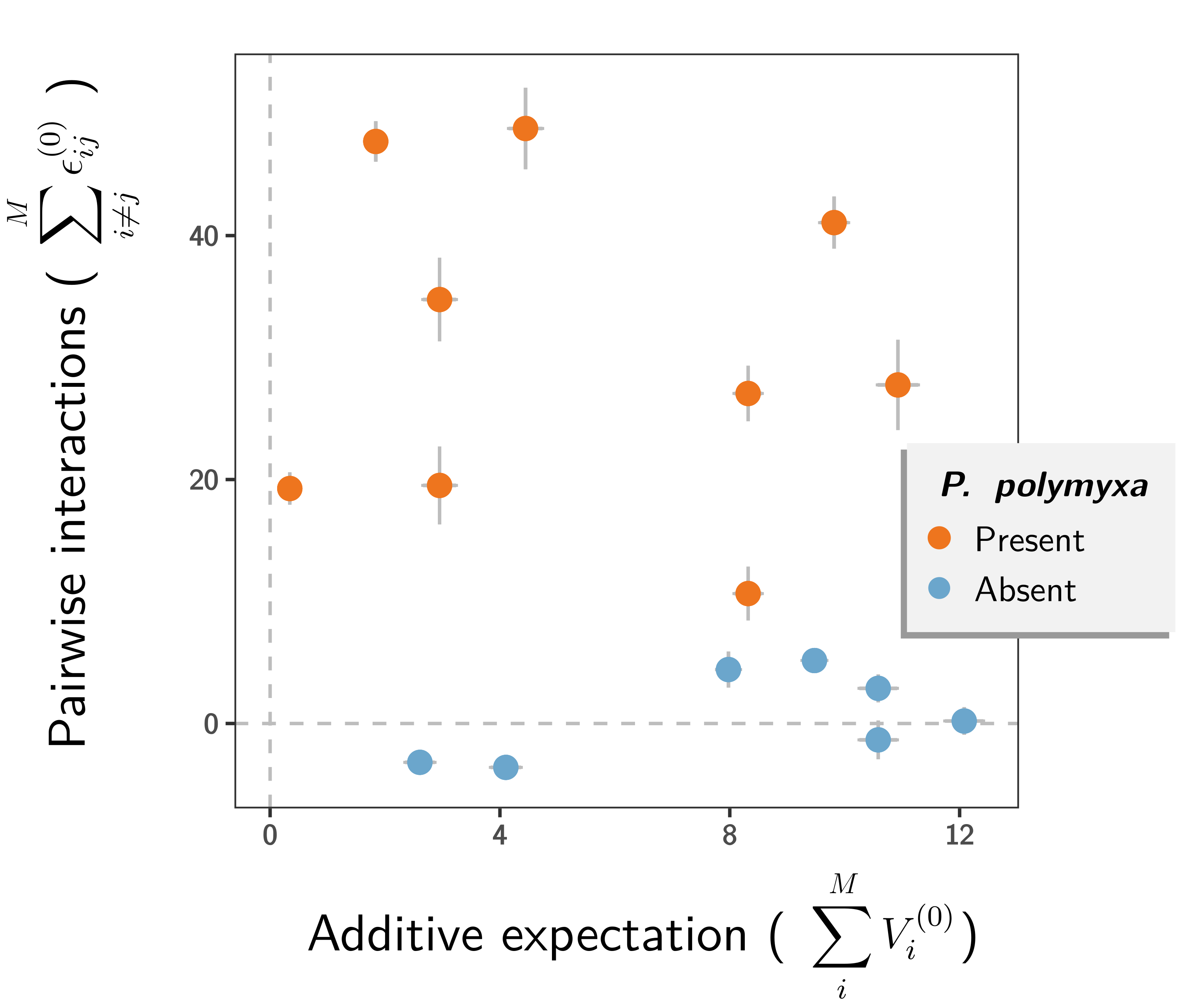

Supplement: S2 Fig — Sum of pairwise interactions (mean ± SE, hr−1) is very weakly correlated to additive expectation (mean ± SE, same units) in three-species communities (ρ = −0.29, P = 0.04). Trios including P. polymyxa are shown in orange, and those not including P. polymyxa are shown in blue. (TIFF) [file pbio.3000550.s002.tiff]

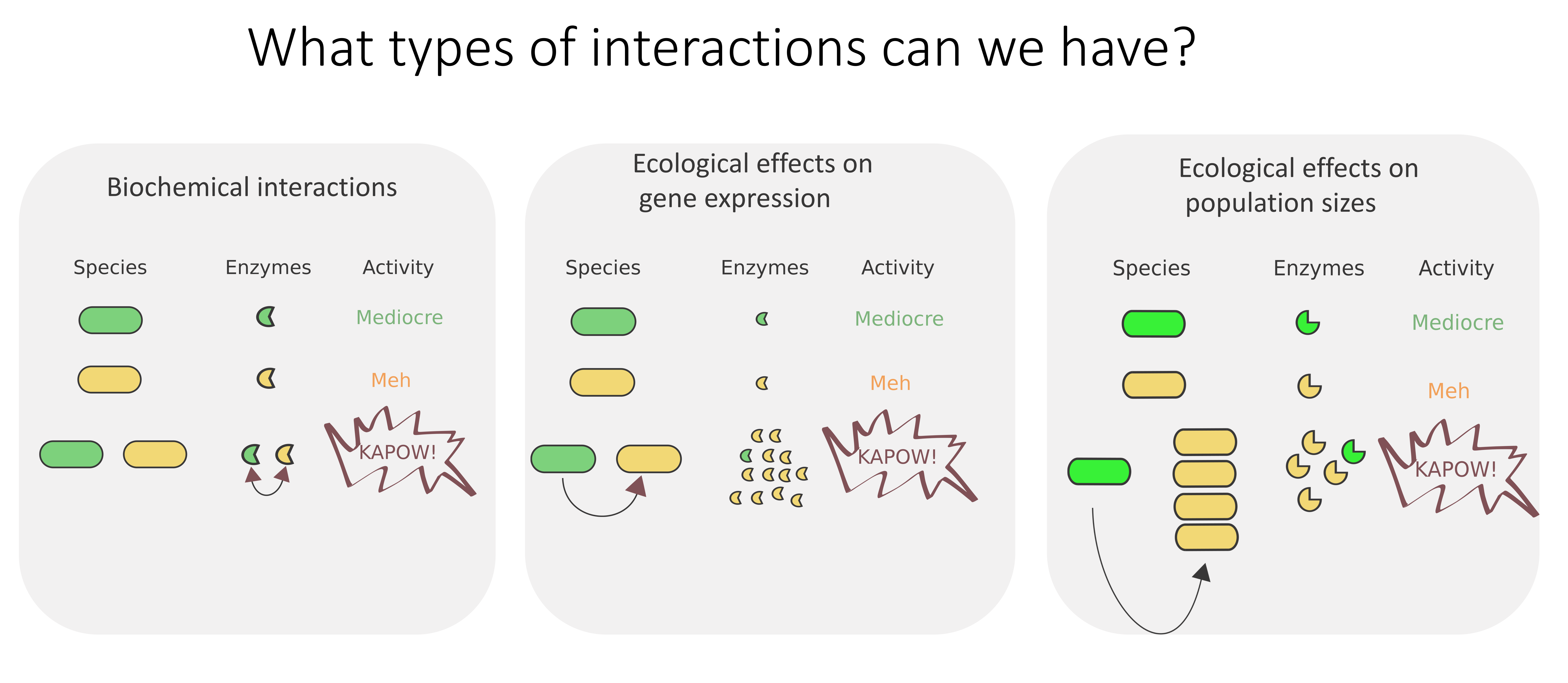

Supplement: S3 Fig — Left panel: Biochemical interactions. The enzymes secreted by different organisms interact with each other producing a higher or lower activity than each would in isolation (e.g., through allosteric interactions or by catalyzing different steps on a complex biochemical pathway). Center panel: Behavioral interactions. Organisms could respond to the presence of other species in their environment by regulating the expression of extracellular enzymes (either inducing or repressing the amount of enzyme produced per cell). Right panel: Population dynamics interactions. Organisms could affect each other’s population size, thereby indirectly affecting the total amount of extracellular enzyme released to the environment. (TIFF) [file pbio.3000550.s003.tiff]

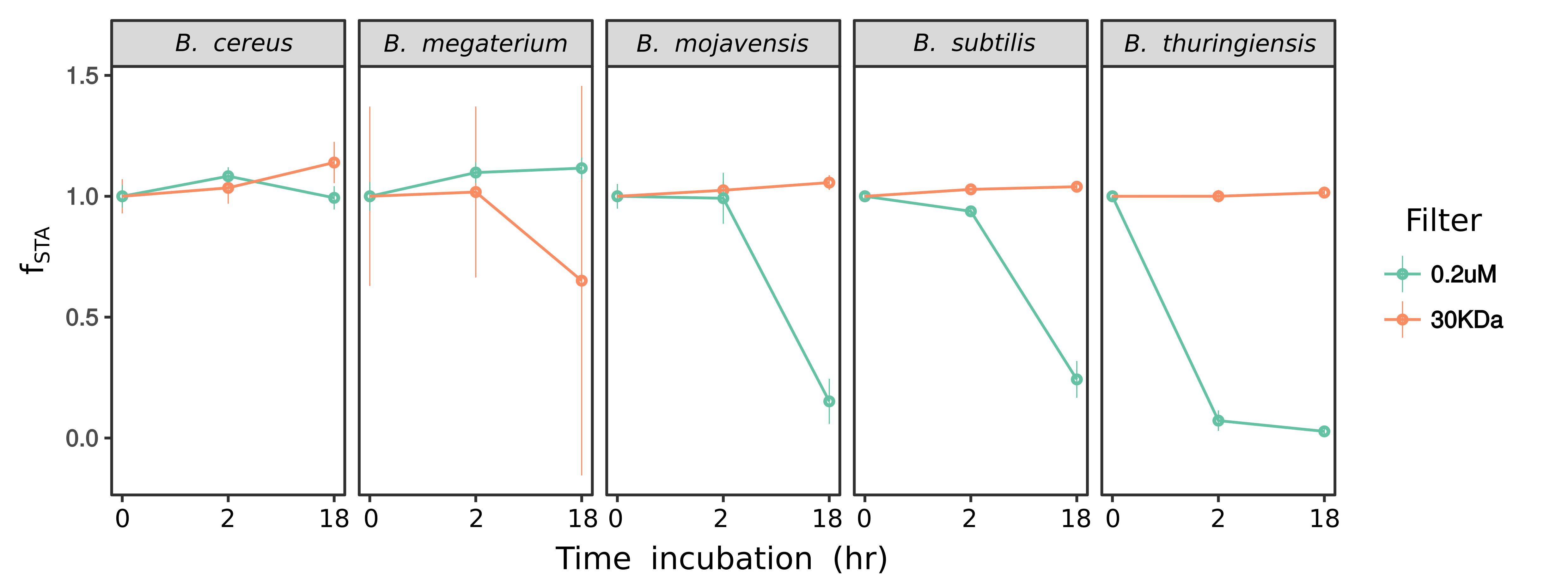

Supplement: S4 Fig — Supernatant from 24-hr cultures in 1x bSAM supplemented with starch were filtered through either 0.2-μM or 30-KDa filters and assayed for amylolytic activity using Lugol staining (Methods) at 0, 2, and 18 hr. Error bars represent ±SD from three replicates. (TIFF) [file pbio.3000550.s004.tiff]

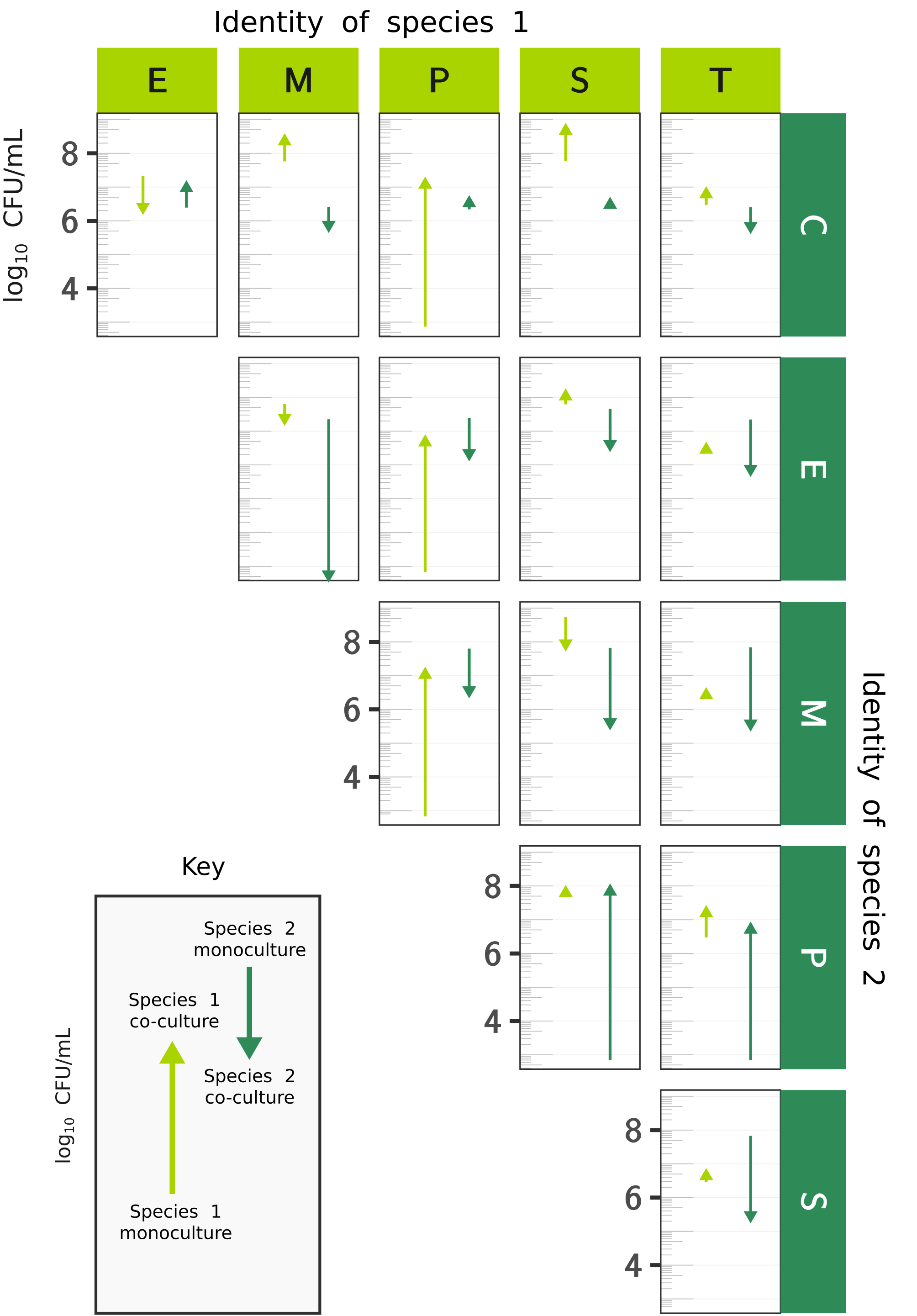

Supplement: S5 Fig — Cultures of the six bacilli species grown at 30°C for 24 hr were concentrated, washed, resuspended in growth media to inoculate monocultures or pairs, and grown at 30°C for an additional 24 hr. Species (light and dark green) are designated as C, B. cereus; E, B. megaterium; M, B. mojavensis; P, P. polymyxa; S, B. subtilis; and T, B. thuringiensis. Upward arrows denote an increase in CFU/mL from the value obtained when the two species are grown in monocultures to the value obtained after the same two species were cocultured for 24 hr. The experiment was done in two biological replicates of which we measured starch concentration in both and counted CFUs in one. Downward arrows indicate a decrease in CFU/mL. CFU, colony forming unit. (TIFF) [file pbio.3000550.s005.tiff]

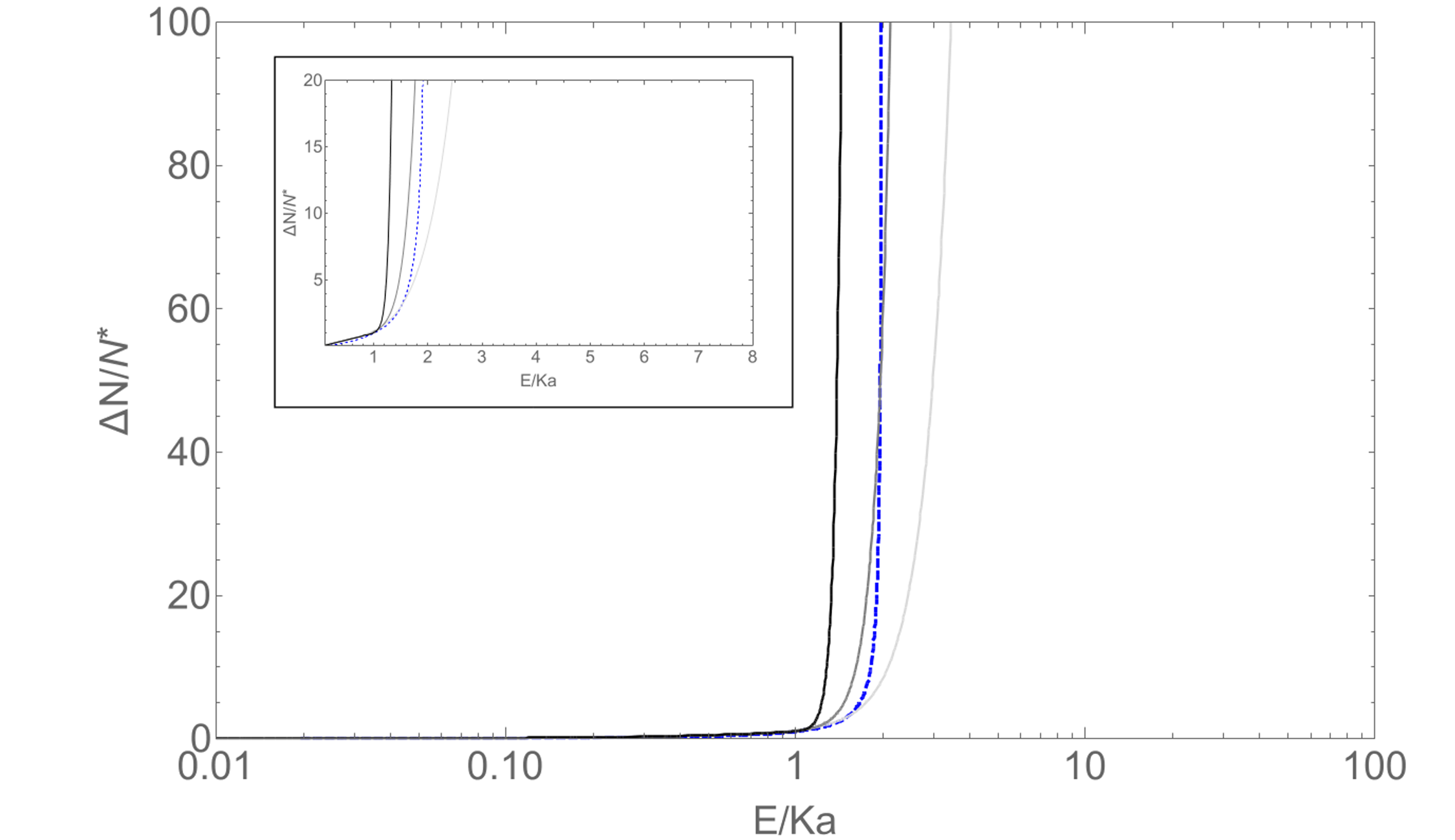

Supplement: S6 Fig — We plot Eq 24 (ΔN/N*=[E/A+1q+1(E/A)q+1]) for various values of q: q = 4 (light gray), q = 8 (dark gray), q = 20 (black). By way of comparison, we plot the inverse of a type II Holling function like the one we use in the paper E/A = (E/A)max ΔN/(N* + ΔN) (dashed blue) for (E/A)max = 2 (the result is not contingent on this choice, and other values gave similar behavior but are not shown for clarity). Inset: log-log scale representation of the same figure. (TIF) [file pbio.3000550.s006.tif]

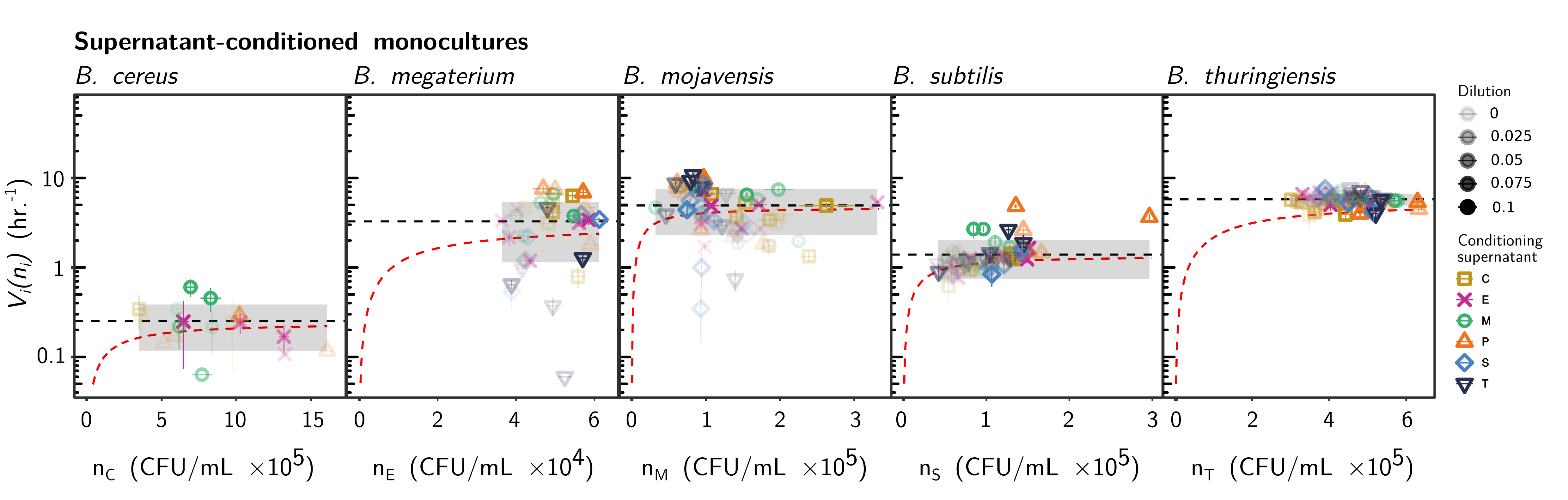

Supplement: S7 Fig — We cultured each of the Bacillus species in 1x bSAM media supplemented with different amounts of filtered spent media from all of the other taxa (the fraction of the final culture volume made up by the spent media ranged between 0 and 0.1, as shown). After 24 hr of culture, the final population size (in CFU/mL) and the amylolytic function V were determined and plotted. With the exception of P. polymyxa (Fig 5C, main text) and B. subtilis, for the whole range of observed population sizes in other Bacilli species the function remained approximately flat, consistently with an underlying saturating function. Thus, for these species, we used the average of the observed Vi(ni) values to estimate parameter ui (dashed black line; gray indicates ±SD), and we conservatively estimated K as one-half of the minimum observed ni in the data shown in these figures. Using V and K parameters estimated in this way, we show the resulting saturating function for each species (dashed red lines). CFU, colony forming unit. (TIFF) [file pbio.3000550.s007.tiff]

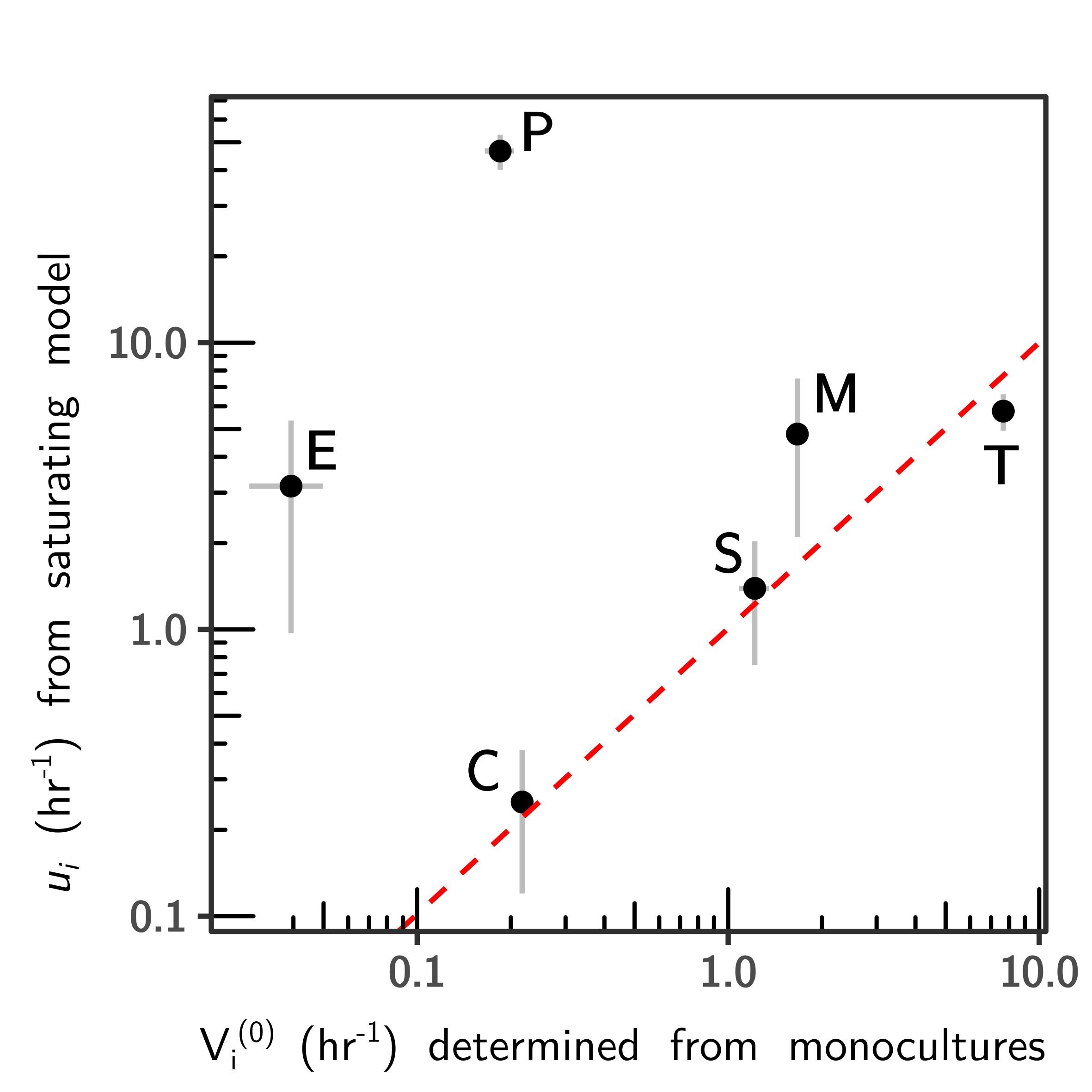

Supplement: S8 Fig — The dashed red line indicates perfect matching. The value of B. mojavensis is likely overestimated, as it reaches higher function values when grown supplemented with spent media of the other species. The value of P. polymyxa is expected not to match, given that Vi(0) is determined in monoculture and in the absence of any facilitation. (TIFF) [file pbio.3000550.s008.tiff]

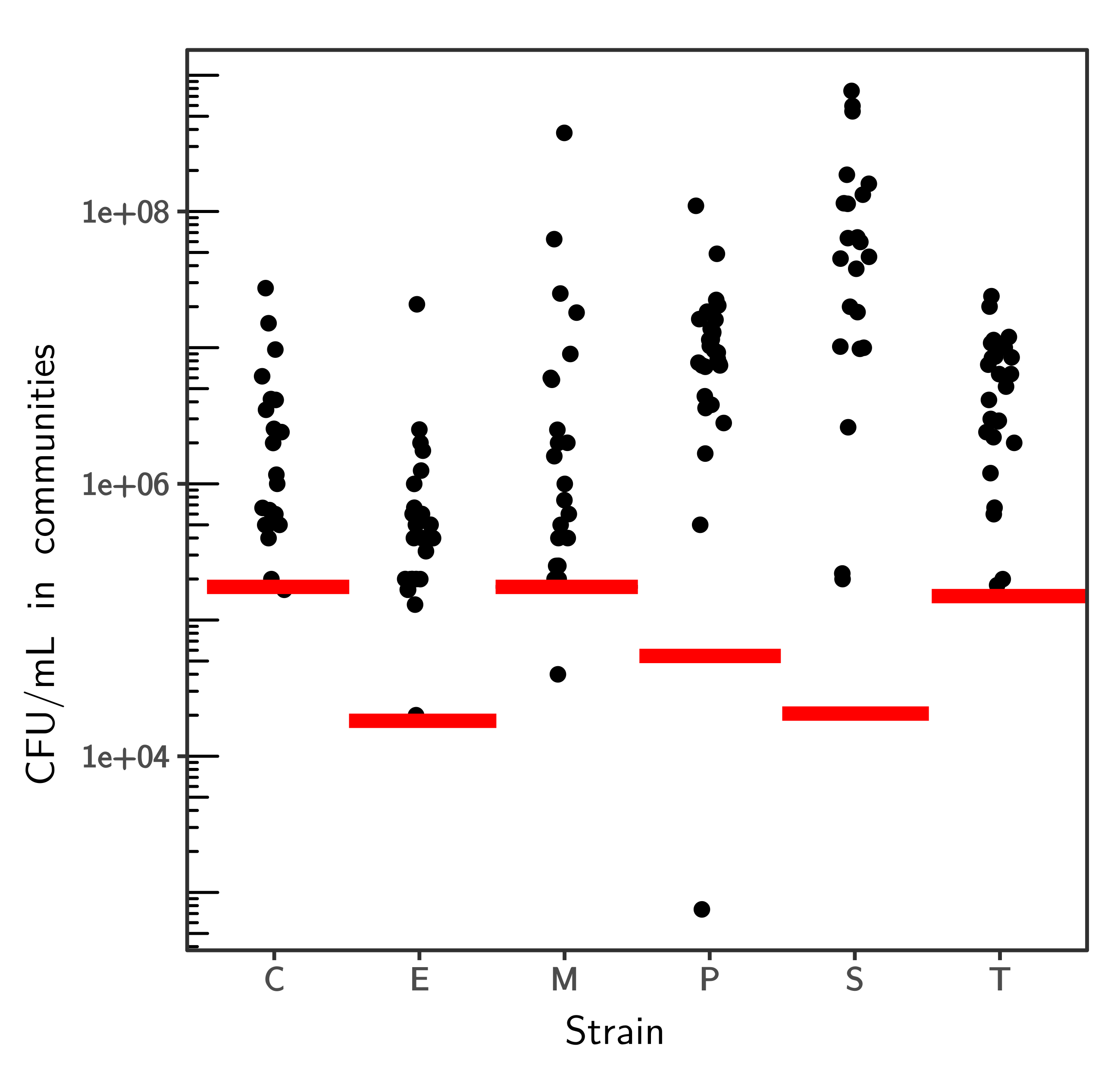

Supplement: S9 Fig — The threshold for each species (red line) was conservatively estimated as one-half of the minimal population size in the supernatant conditioning experiment (Figs 5C and S7). The black points represent the CFU/mL counts determined for each species in all of the consortia that it is present. CFU, colony forming unit. (TIFF) [file pbio.3000550.s009.tiff]

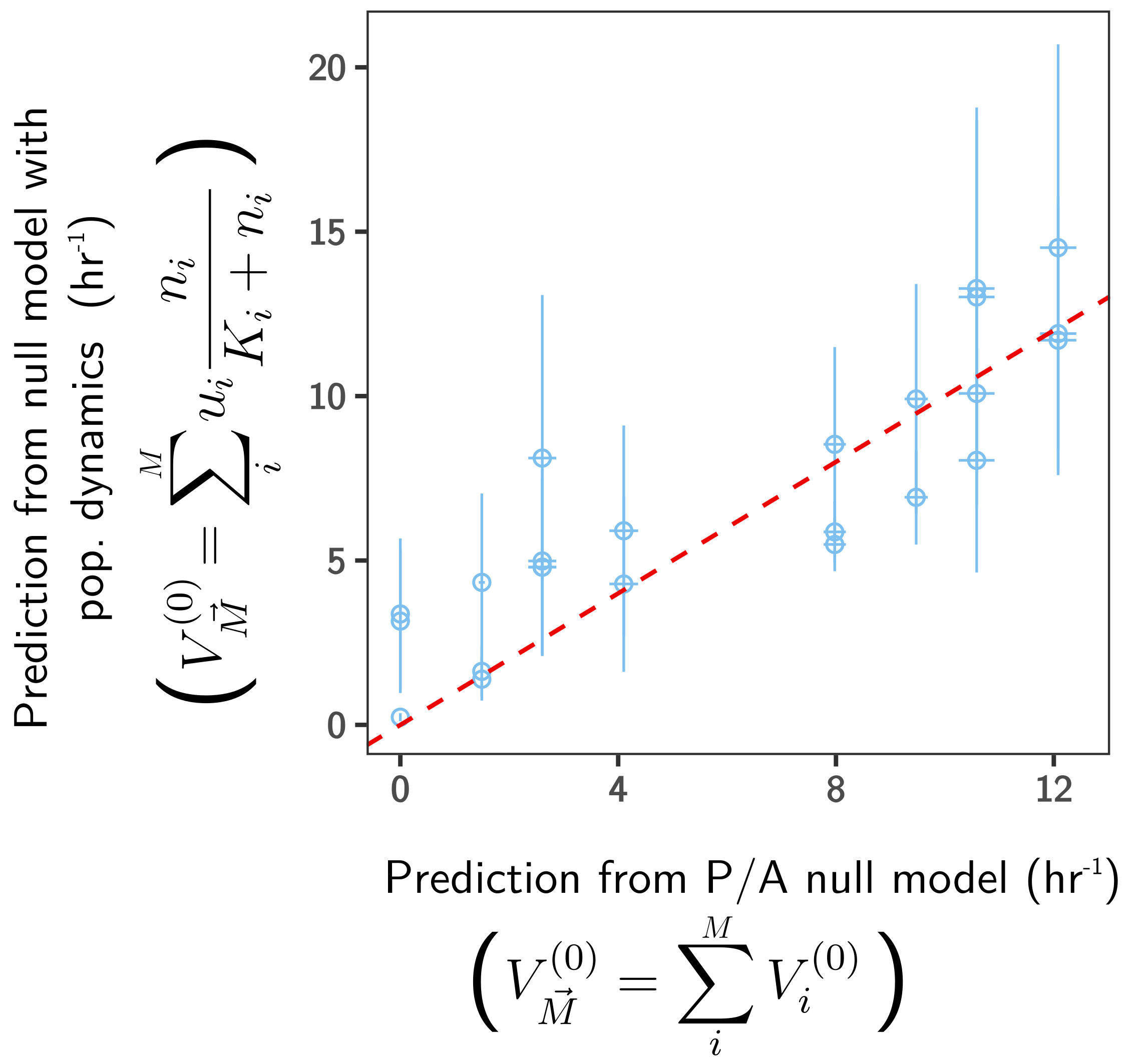

Supplement: S10 Fig — Each point represents one consortium; error bars represent ±SE. (TIFF) [file pbio.3000550.s010.tiff]

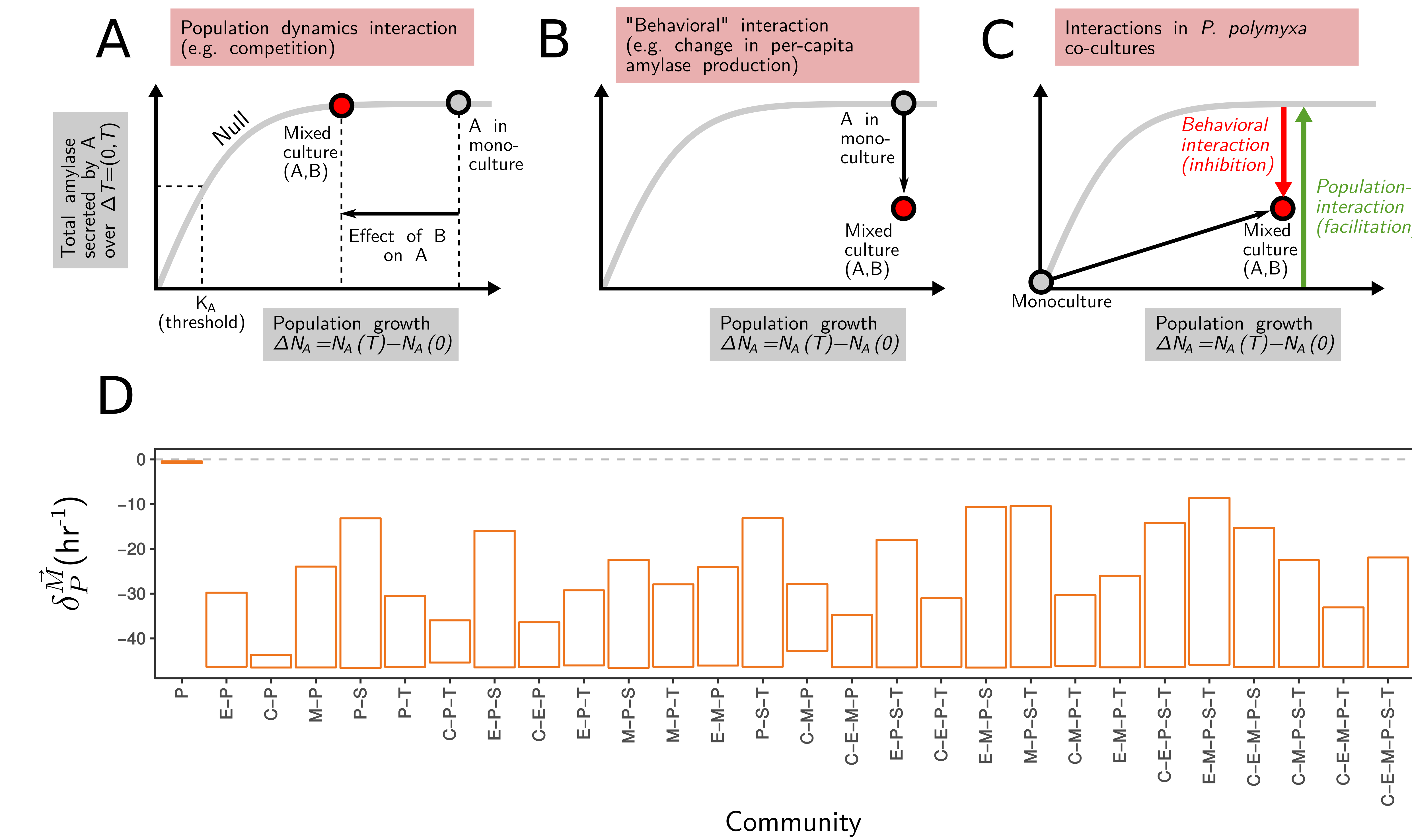

Supplement: S11 Fig — We depict the effect of species B on the amount of enzyme released by species A at different population sizes: (A) An example of how population dynamics interactions may affect the amount of amylase produced by a species: when species B competes with species A, it lowers its population size, but it does not alter the amount of amylase it expresses relative to monoculture. (B) An example of how behavioral interactions may affect the amount of amylase produced by a species: Species B does not affect the growth of species A, but the latter responds to the presence of species B by lowering its investment in amylase production. (C) We propose that the function of communities containing P. polymyxa could be explained by a combination of growth facilitation, which pushes the population size over the threshold KP, and a behavioral interaction in which P. polymyxa reduces its investment in amylase production in coculture with other species. (D) We estimate the lower and upper bound inhibition of the contribution of P. polymyxa to community function in mixed culture with other species (i.e., δPM→). It is straightforward to show that δPM→∈[-uPnPnP+KP,VM→(0)-uPnPnP+KP] where, as defined in the text, uP is the activity of P. polymyxa at saturating population size (nP>>KP), and VM→(0) is the activity of the consortia. The lower bound corresponds to P. polymyxa completely suppressing its contribution, and the higher bound corresponds to all other species suppressing their contribution while P. polymyxa is the only one secreting amylase. (TIFF) [file pbio.3000550.s011.tiff]

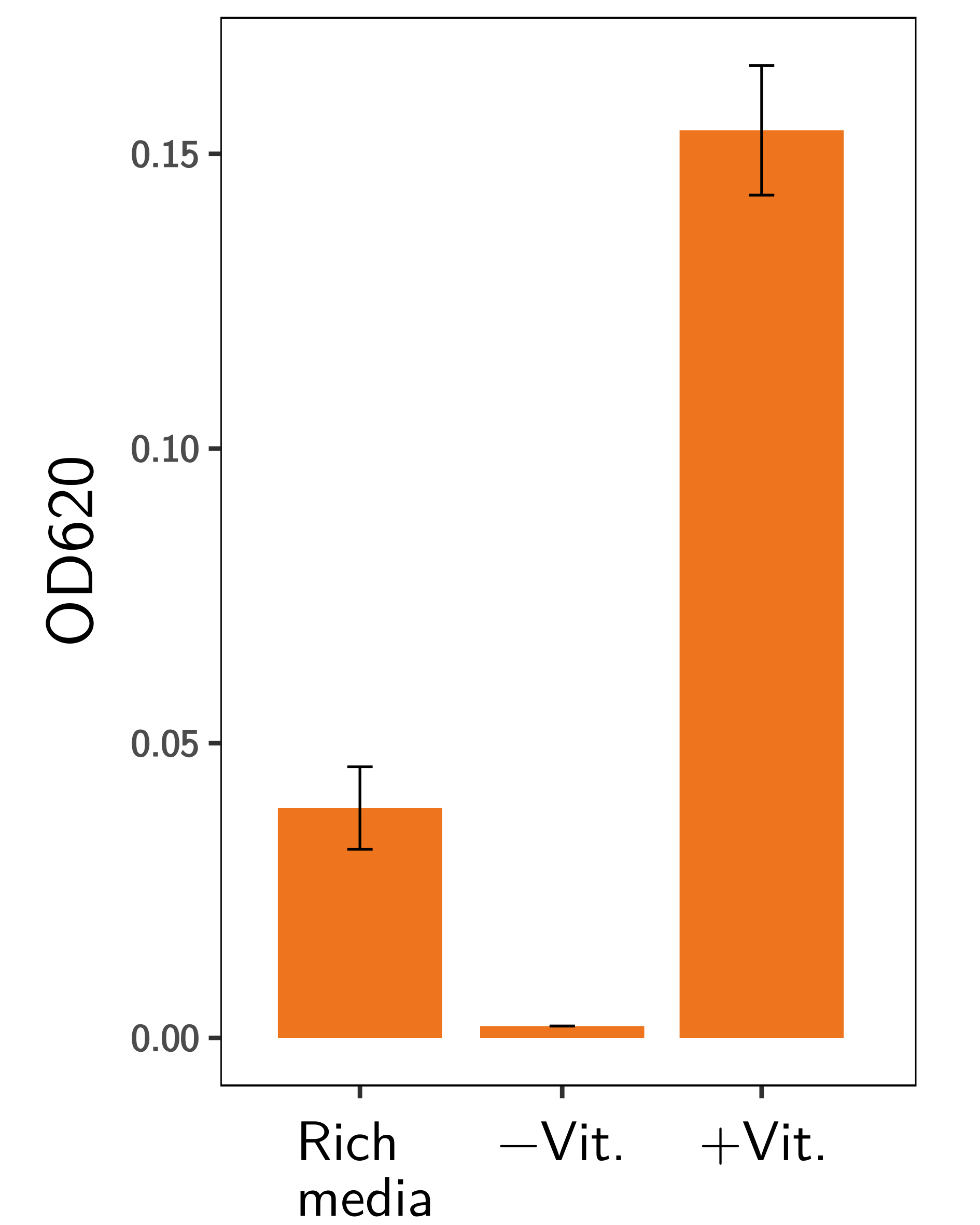

Supplement: S12 Fig — Optical density at 620 nm of P. polymyxa cultures grown at 30°C for 24 hr in rich media (beef extract + 2% w/v starch) or 1× bSAM supplemented or not, as indicated, with 1% vitamin supplement (v/v, ATCC MD-VS). Note that the non-vitamin-supplemented condition (“- Vitamins”) would correspond to the same growth medium (1× bSAM) used in all of the experiments in this paper. This medium (1× bSAM) does not contain vitamins. As expected from prior work [66], addition of a biotin-containing vitamin supplement to bSAM (“+Vitamins”) is sufficient for P. polymyxa to grow, as expected. Plots show the media for two replicates and the standard error. (TIFF) [file pbio.3000550.s012.tiff]

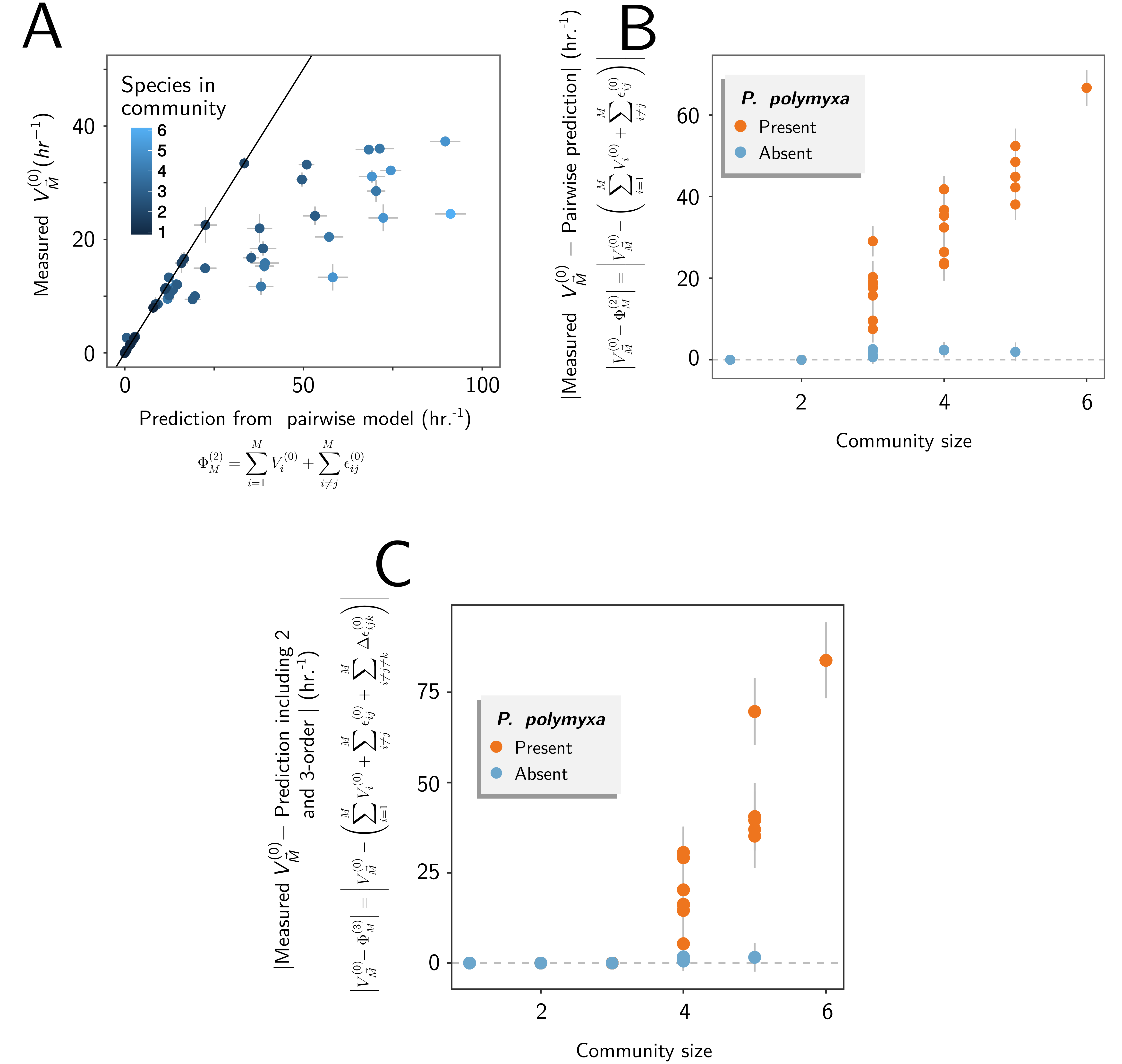

Supplement: S13 Fig — (A) Plot showing the prediction of the function (V) from a model that includes only up to pairwise interactions against the experimentally measured value for consortia of different richness (represented by point color). (B) Strength of higher-order interactions, measured as the absolute value of the difference between the function determined experimentally (amylolytic activity VM→(0), hr−1) and the expected one using the model including single and pairwise effects. (C) The strength of high-order interactions increases with community size also when accounting for third-order interactions. Similarly to (B), interactions of order higher than three are measured here as the absolute value of the difference between the function determined experimentally (Vi(0), hr−1) and the expected one using the model including only single, pairwise, and third-order effects. Orange and blue dots represent communities with or without P. polymyxa, respectively. All error bars represent ±SE. (TIFF) [file pbio.3000550.s013.tiff]

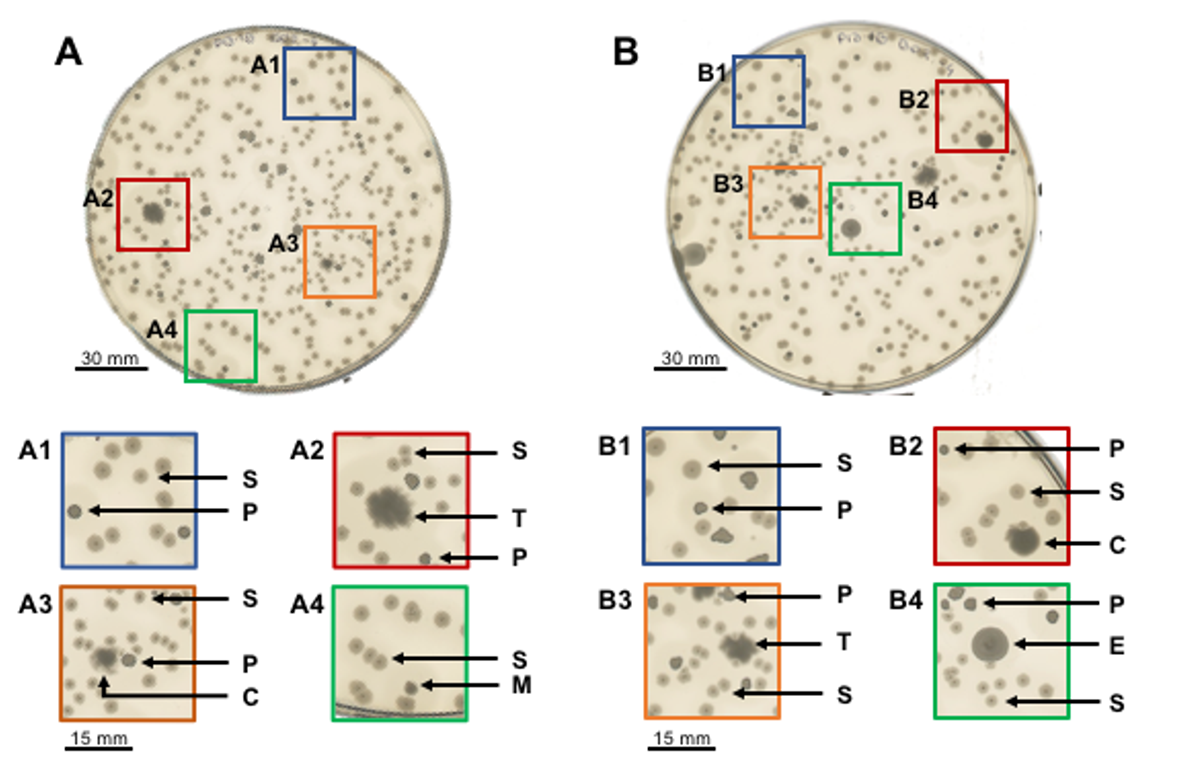

Supplement: S14 Fig — Combinatorially assembled communities were incubated in 1x bSAM at 30°C for 24 hr and stored at −80°C in 40% glycerol. Approximately 20 μl of the frozen stock was melted and serially diluted 1:10 up to 1:105. Fifty microliters of dilutions 1:104 was plated onto BE Starch and incubated at 30°C for 48 hr. To record colony growth and development, plates were scanned every 24 hr with an EPSON Perfection V700-V750 scanner at a 300-dpi resolution. Colonies were discriminated by naked eye using a Laxco MZS1 Series Stereo Zoom Microscope or the scanned images according to their color, shape, size, roughness of borders, and size of the halo produced upon starch degradation. (A) Agar plate showing the community composed of B. cereus, B. mojavensis, P. polymyxa, B. subtilis, and B. thuringiensis. Insets A1–A4 have been scaled up to show the different colony morphologies found. Arrows point to each of the five Bacillus species in this consortium. (B) Agar plate showing the community composed of B. cereus, B. megaterium, P. polymyxa, B. subtilis, and B. thuringiensis. Insets B1–B4 have been scaled up to show the different colony morphologies found. Arrows point to each of the five Bacillus species in this consortium. Species are designated as C, B. cereus; E, B. megaterium; M, B. mojavensis; P, P. polymyxa; S, B. subtilis; and T, B. thuringiensis. (TIF) [file pbio.3000550.s014.tif]
